# Group-I PAKs-mediated phosphorylation of HACE1 at serine 385 regulates its oligomerization state and Rac1 ubiquitination

**DOI:** 10.1038/s41598-018-19471-2

**Published:** 2018-01-23

**Authors:** Maria I. Acosta, Serge Urbach, Anne Doye, Yuen-Wai Ng, Jérôme Boudeau, Amel Mettouchi, Anne Debant, Edward Manser, Orane Visvikis, Emmanuel Lemichez

**Affiliations:** 1 C3M, Inserm, Equipe labellisée Ligue contre le Cancer, University of Côte d’Azur, Nice, F-06204 France; 2 FPP at IGF, CNRS, Inserm, University of Montpellier, Montpellier, F-34094 France; 3 CRBM, CNRS, University of Montpellier, Montpellier, F-34293 France; 4 sGSK Group, IMCB, A*STAR, Singapore, 138673 Singapore

## Abstract

The regulation of Rac1 by HACE1-mediated ubiquitination and proteasomal degradation is emerging as an essential element in the maintenance of cell homeostasis. However, how the E3 ubiquitin ligase activity of HACE1 is regulated remains undetermined. Using a proteomic approach, we identified serine 385 as a target of group-I PAK kinases downstream Rac1 activation by CNF1 toxin from pathogenic *E*. *coli*. Moreover, cell treatment with VEGF also promotes Ser-385 phosphorylation of HACE1. We have established *in vitro* that HACE1 is a direct target of PAK1 kinase activity. Mechanistically, we found that the phospho-mimetic mutant HACE1(S385E), as opposed to HACE1(S385A), displays a lower capacity to ubiquitinate Rac1 in cells. Concomitantly, phosphorylation of Ser-385 plays a pivotal role in controlling the oligomerization state of HACE1. Finally, Ser-385 phosphorylated form of HACE1 localizes in the cytosol away from its target Rac1. Together, our data point to a feedback inhibition of HACE1 ubiquitination activity on Rac1 by group-I PAK kinases.

## Introduction

E3 ubiquitin ligases (E3s) are critical gatekeepers of cell homeostasis^1^. While we have begun to appreciate their structure and the diversity of their targets, we fall short on knowledge about their general integration in cell signaling. One essential question in particular is to better understand the cross-talk between kinases and ubiquitin ligases^2^. This is particularly true for HACE1, which is a critical regulator of active Rac1 flux for which we still lack identified regulators^3^.

Rac1, together with Cdc42 and RhoA, are the most extensively studied members of the Rho GTPase family, which are intracellular signaling proteins that control a variety of cellular processes, such as actin remodeling and transcription^4^. Rho GTPases act as molecular switches that cycle between an inactive form bound to GDP and an active form bound to GTP. In response to various environmental stimuli, inactive Rho proteins are charged with GTP, which induces conformational changes allowing Rho proteins to bind to effector proteins. These effectors, in turn, either relay or directly execute cellular responses driven by the Rho-activating environmental stimuli. More than 100 effector proteins of Rho GTPases have been described; some correspond to scaffold proteins, while others harbor enzymatic activities, notably kinases^4^. The first identified and best characterized effectors activated by Rac1 are the family of P-21 Activated serine/threonine Kinases (PAKs)^5^. In mammals, the PAK family consists of six members classified into two groups. Rac1 and Cdc42 activate group-I, which comprises PAK1, PAK2 and PAK3. Group-II, which comprises PAK4, PAK5 and PAK6, are only regulated by Cdc42^6^. Group-I PAKs are highly homologous but show different profiles of tissue expression. While PAK2 is found in virtually all tissues, PAK1 and PAK3 display a more restricted expression patterns; PAK1 is expressed in the mammary gland, muscle, spleen and brain tissues, while PAK3 expression is restricted to the brain^7^. Genetics studies have established that PAKs play a significant role in tissue development and that PAK expression deregulation is linked to cancer progression^8,9^.

The regulated activity of Rac1 allows the integration of the many signals involved in the maintenance of cell homeostasis and cellular dynamics. The GDP/GTP cycle is controlled by the following regulatory proteins: (i) Guanine nucleotide Exchange Factors (GEFs), which facilitate the exchange of GDP and GTP; (ii) GTPase activating Proteins (GAPs), which increase the intrinsic rate of GTP hydrolysis; and (iii) RhoGDIs, which sequester Rho proteins in the cytosol. Additionally, the control of active Rac1 flux by the E3 ubiquitin ligase HACE1 is emerging as an important aspect of Rac1 signaling^3^. We and others have shown that HACE1 ubiquitinates Rac1 once it is activated, either by using point mutants (Q61L, Q61E, and G12V), by over-expressing the GEF domain of Dbl, or in response to growth factors such as Hepatocyte Growth Factor (HGF), Epidermal Growth Factor (EGF) and Heregulin (HRG)^3,10,11^. The regulation of Rac1 by ubiquitination was first revealed in cells intoxicated by Cytotoxic Necrotizing Factor 1 (CNF1)^12^, which is produced by pathogenic strains of *E*. *coli* from phylogenetic group B2. After endocytosis into the host cells, the toxin CNF1 translocates its catalytic domain into the cytosol, where it deamidates RhoA glutamine residue Q63 (Q61 in Rac1 and Cdc42) into a glutamic acid^13,14^. Because this residue is essential for the GTP hydrolysis, its deamidation by CNF1 impairs the GTPase activity of Rho proteins and locks them in a GTP-bound state. As a consequence of its permanent activation, Rac1 gets ubiquitinated by HACE1 and is subsequently targeted by the 26 S proteasome degradation machinery^3,12^.

HACE1 is an important tumor suppressor whose expression is lost in a variety of human cancers, including Wilm’s tumor, B-cell lymphoma, and colorectal, gastric and breast cancers^11,15–19^. The repression of HACE1 expression has been shown to be a consequence of epigenetic silencing or chromosomal alterations^15–19^. A major demonstration of HACE1 tumor suppressor activity came a decade ago from the observation that *hace1* KO mice develop spontaneous late-onset cancers from the three germ-layers^16^. Since then, several studies have converged to the idea that HACE1 is a guardian of cell homeostasis by controlling ROS levels and the autophagy of protein aggregates^20–22^. Moreover, HACE1 also controls cell growth, migration and invasion, which are key features of cancer progression^10,11^. Interestingly, the deregulation of Rac1 ubiquitination due to the loss of HACE1 contributes to higher NADPH-dependent ROS production, which leads to DNA damage and cell hyper-proliferation^20^. Additionally, a recent study has revealed that HACE1-induced ubiquitination of Rac1 in mammary gland epithelial cells (MCF12A) plays a major protective role against HER2/Neu-mediated breast tumorigenesis^11^. More recently, several cancer-associated missense mutations in the *hace1* gene that inhibit Rac1 ubiquitination and impair cell growth have been identified, indicating that HACE1 activity can be altered in cancer^23^.

Despite its importance in cell homeostasis, nothing is known about HACE1 regulation at the post-translational level. HACE1 possesses an N-terminal ankyrin-repeat domain (ANK) and a C-terminal catalytic HECT domain. The ANK and HECT domains are separated by a Middle region (MID) that does not harbor any structural homology with other known domains. Several phospho-proteomics studies have identified residues in HACE1 that are phosphorylated^24^. Nevertheless, the context of these post-translational modifications and their consequences on the activity of this essential regulator remain to be defined. Here, we show the essential roles of group-I PAK kinases on the phospho-regulation of HACE1 E3 ubiquitin ligase activity and its oligomerization state.

## Results

### CNF1 increases HACE1 Ser-385 phosphorylation

**Figure 1:**
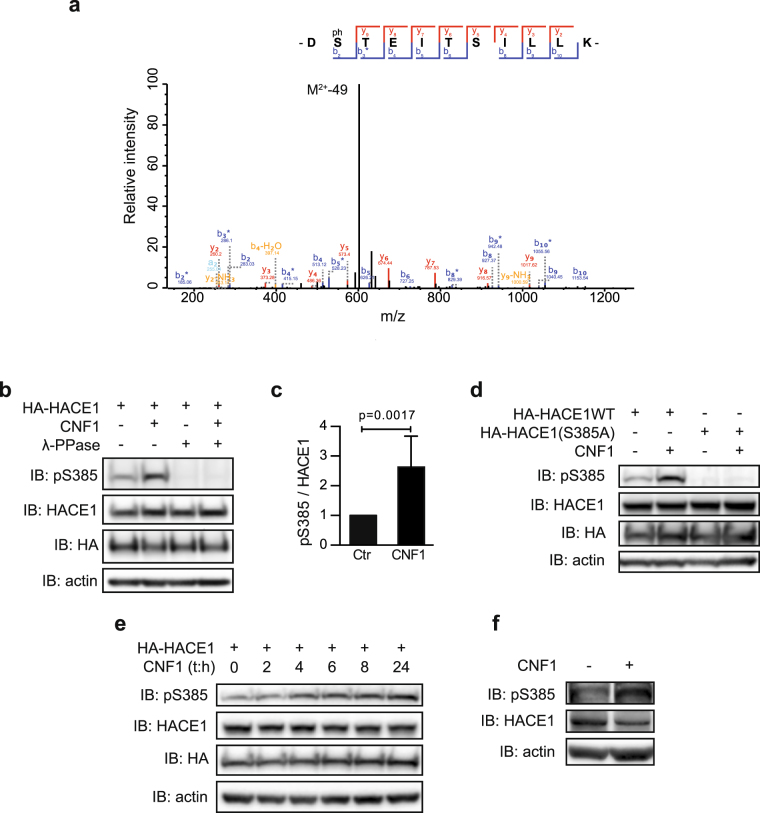
CNF1 increases phosphorylation of HACE1 on Ser-385. (**a**) Fragmentation spectra of the DS(p)TEITSILLK(+2) peptide showing that Ser-385 is phosphorylated. (**b**) Protein lysates from HUVECs transfected with HA-HACE1(WT) and treated with CNF1 at 10^−9^ M for 24 hours were treated or not with λ-phosphatase (λ-PPase) and analyzed by immunoblot (IB) using the indicated antibodies. IB: actin is used as a loading control. (**c)** Graph showing levels of pSer-385HACE1 relative to HACE1 total protein levels quantified by densitometry from the IB analysis. Data correspond to the mean ± SD of >3 biological replicates. p value was determined by one-sample t-test. (**d)** Protein lysates from HUVECs transfected with HA-HACE1(WT) or HA-HACE1(S385A), treated with CNF1 at 10^−9^ M for 24 hours and analyzed by IB. (**e)** Protein lysates from HUVECs transfected with HA-HACE1(WT), treated with CNF1 at 10^−9^ M for the indicated times, and analyzed by IB. (**f)** Protein lysates from HUVECs treated with CNF1 at 10^−9^ M for 24 hours. IBs in (**b**,**d**,**e**,**f**) are cropped from the full-length blots shown in Supplemental Figure S9.

To explore the possible cross-talk between Rho GTPases and HACE1, we conducted a study aimed at characterizing the phosphorylation status of HACE1 upon activation of Rho GTPases. We undertook an unbiased proteomics approach to identify HACE1 amino acid residues that were differentially phosphorylated in cells treated with the Rho-activating toxin CNF1. This was performed by tandem mass spectrometry (MS/MS) comparative analysis of trypsin-digested immuno-purified HA-HACE1 wild-type (WT) from primary Human Umbilical Vein Endothelial Cells (HUVECs) that were either untreated or treated with CNF1 for 24 hours. Following MS/MS analysis, we performed a database search using phosphorylation as a variable modification. This allowed the identification of 5 peptides corresponding to two phosphorylated residues: Ser-337 and Ser-385 (Fig. 1a and Supplementary Figure S1a–d). Quantification of phospho-peptide occurrences in CNF1-treated cells compared to control cells revealed a specific increase of Ser-385-containing peptides with a cut off of >2-fold (Supplementary Figure S1e).

To validate these results and to study the phospho-modulation of HACE1, we generated a Ser-385 and a Ser-337 phospho-specific polyclonal antibodies (referred to as pS385 and pS337) and measured the levels of phosphorylated HACE1 by immunoblot assays. In agreement with the proteomics analysis, we found that CNF1 treatment of HUVECs expressing HACE1 leads to a significant increase in Ser-385 phosphorylation levels compared to control cells but doesn’t significantly impact the phosphorylation levels of Ser-337 (Fig. 1b and c and Supplementary Figure S1f and g). To ascertain the specificity of the pS385 antibody towards phosphorylated HACE1, protein extracts were incubated with λ-phosphatase. As shown in Fig. 1b, signals detected using the pS385 antibody disappeared after λ-phosphatase treatment, indicating that the pS385 antibody specifically recognizes the phosphorylated form of HACE1. Additionally, we found that the pS385 antibody detects HACE1(WT) but not the phospho-resistant mutant HACE1(S385A), indicating that the pS385 antibody specifically recognizes the phosphorylated Ser-385 in this context (Fig. 1d). The kinetics of HACE1 Ser-385 phosphorylation in CNF1-treated cells showed an increase in phosphorylation of up to 24 hours (Fig. 1e). Even though endogenous HACE1 is expressed at very low levels in HUVECs, we detected the increase in endogenous HACE1 phosphorylation on Ser-385 in CNF1-treated cells (Fig. 1f). Collectively, our data establish that HACE1 phosphorylation at Ser-385 is more abundant in cells treated with CNF1 than in control cells.

### Rac1/Cdc42 controls HACE1 Ser-385 phosphorylation

**Figure 2:**
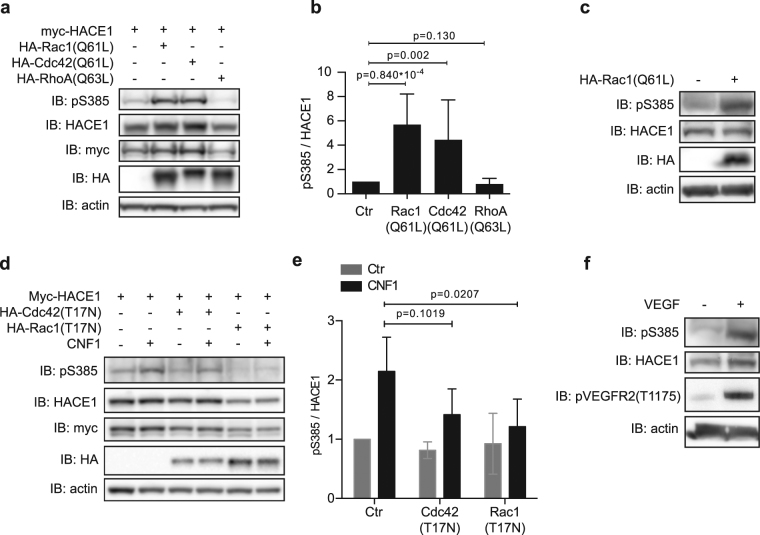
Activation of Rac1 or Cdc42 mediates the phosphorylation of HACE1 at Ser-385. (**a**) Protein lysates from HUVECs transfected with myc-HACE1(WT) together with HA-tagged active mutant of Rho-GTPases were analyzed by immunoblot (IB) using the indicated antibodies. IB: actin is used as a loading control. (**b**) Graph showing levels of pS385 relative to HACE1 total protein levels quantified by densitometry from the IB analysis described in A. Data correspond to the mean ± SD of 3 biological replicates. p values were determined by unpaired one-sample t-test. (**c)** Protein lysates from HUVECs transfected with HA-Rac1(Q61L) and analyzed as in (**a**). (**d**) Protein lysates from HUVECs transfected with HA-HACE1(WT) alone or together with dominant negative myc-Rac1(T17N) or myc-Cdc42(T17N), treated with CNF1 at 10^−9^ M for 24 hours and analyzed as in (**a**). (**e**) Graph showing levels of pS385 relative to HACE1 total protein levels quantified by densitometry from the IB analyses described in (**d**). Data correspond to the mean ± SD of 3 biological replicates. p values were determined by unpaired two-sample t-test. (**f**) Protein lysates from HUVECs treated with VEGF 20ng/ml for 10 min and analyzed as in (**a**). IBs in (**a**,**c**,**d**,**f**) are cropped from the full-length blots shown in Supplemental Figure S10.

Because CNF1 triggers the constitutive activation of several Rho GTPases^13,14^, we assessed whether HACE1 phosphorylation in CNF1-treated cells is mediated by Rho GTPase activation. Using the pS385 antibody, we first analyzed the levels of HACE1 phosphorylation on Ser-385 in HUVECs co-expressing active forms of Rac1, Cdc42 and RhoA. The expression of both Rac1(Q61L) and Cdc42(Q61L) induced higher levels of Ser-385 phosphorylation compared to control conditions (Fig. 2a,b). Conversely, the expression of RhoA(Q63L) had no effect (Fig. 2a,b). The expression of Rac1(Q61L) also induced a clear increase in endogenous HACE1 phosphorylation on Ser-385 (Fig. 2c). Over-expression of the dominant negative forms Rac1(T17N) or Cdc42(T17N) before CNF1 treatment revealed the major role of Rac1 in the CNF1 signaling leading to HACE1 phosphorylation of Ser-385 (Fig. 2d,e). Altogether, these results show that CNF1 treatment promotes the phosphorylation of HACE1 on Ser-385 in a Rac1-dependent manner in HUVECs. To determine whether phosphorylation of HACE1 can also be induced by physiological activation of Rac1, we treated HUVEC cells with vascular endothelial growth factor (VEGF), a potent activator of Rac1^25^. Remarkably, this revealed that VEGF treatment strongly induces phosphorylation of endogenous HACE1 on Ser-385, indicating that HACE1 phosphorylation on Ser-385 occurs in response to a physiological stimulus (Fig. 2f).

We then sought to determine whether this phosphorylation of HACE1 is specific to endothelial cells or whether it can be observed in other cell types. We analyzed the phosphorylation of HACE1 in mammary gland epithelial MCF12A cells, a cell type in which the HACE1/Rac1 signaling axis is functional^11^. Interestingly, we found that CNF1 treatment or the expression of Rac1(Q61L) greatly increased the phosphorylation of Ser-385 in MCF12A cells (Supplementary Figure S2a,b). This result shows that the induction of HACE1 phosphorylation on Ser-385 is not restricted to endothelial cells and might be a broader mechanism of HACE1 regulation. Altogether, these results show that CNF1 and Rac1 induce HACE1 phosphorylation on Ser-385 in various cell types.

### Group-I p-21 activated kinases phosphorylate HACE1 on Ser-385

**Figure 3:**
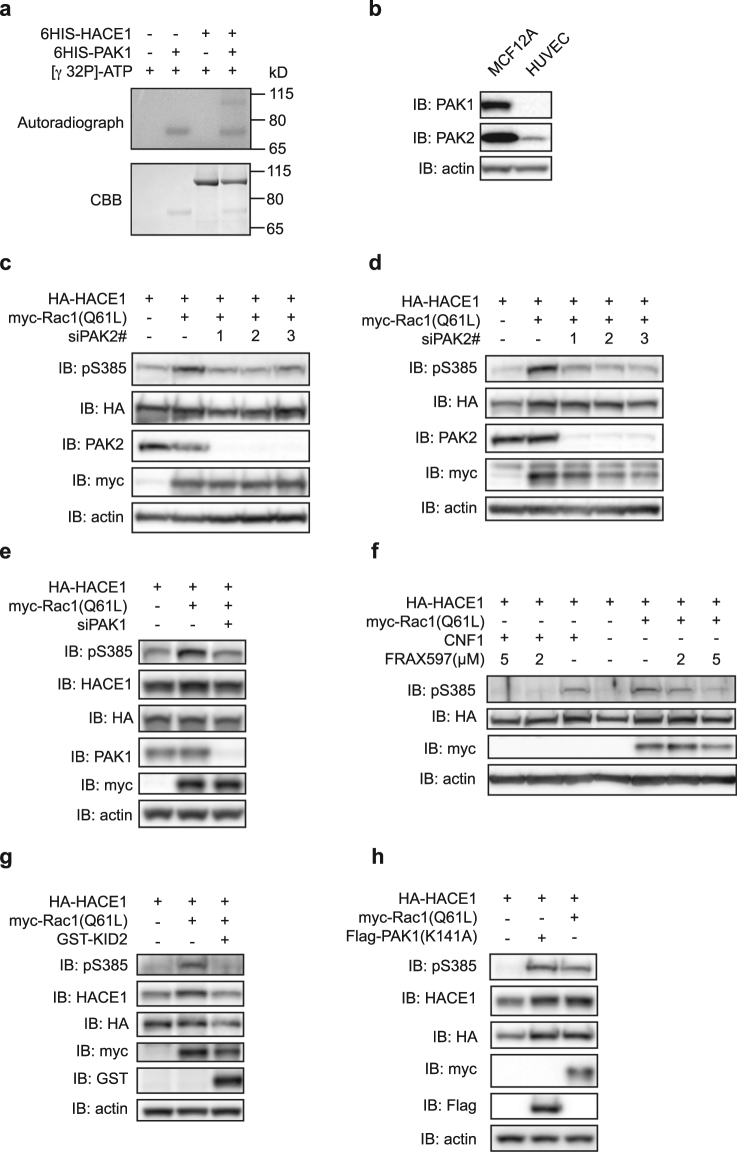
Group-I PAKs induce direct phosphorylation of HACE1. (**a**) *In vitro* [γ ^32^P]-ATP kinase assay using recombinant 6His-HACE1 and recombinant 6His-PAK1 analyzed by autoradiography and Coomassie Brilliant Blue (CBB) staining. (**b**) MCF12A and HUVEC protein lysates analyzed by immunoblot (IB) using anti-PAK1 and anti-PAK2 antibodies. IB: actin is used as a loading control. (**c–e**) Protein lysates from (**c**) HUVECs and (**d,e**) MCF12A cells transfected with siRNAs targeting (**c**,**d**) PAK2 or (**e**) PAK1 and plasmid expressing HA-HACE1 and myc-Rac1(Q61L) and analyzed by IB using the indicated antibodies. (**f**) Protein lysates from MCF12A cells transfected with HA-HACE1, either intoxicated with CNF1 for 16 hours or co-transfected with myc-Rac1(Q61L), and treated with FRAX597 at the indicated concentration for 16 hours before IB analysis. (**g**,**h**) Protein lysates from MCF12A cells transfected with HA-HACE1, myc-Rac1(Q61L) and GST-KID2 or Flag-PAK1K141A and analyzed by IB. IBs in (**a**–**h**) are cropped from the full-length blots shown in Supplemental Figure S11.

We then sought to determine the kinases responsible for the phosphorylation of HACE1 on Ser-385. Two independent phospho-proteomics screens performed in HEK293 cells have previously identified the phosphorylation of Ser-385 in HACE1 but found that it is independent of Protein Kinase D1 (PKD1) or mammalian Target of Rapamycin (mTOR) activity^26,27^. Accordingly, inhibition of mTOR using rapamycin or Torin1 had no impact on the level of Ser-385 phosphorylation in Rac1(Q61L)-expressing cells (Supplementary Figure S3). Moreover, we found equal levels of Ser-385 phosphorylation in control and PKD1-depleted cells (data not shown). To narrow down the potential kinases responsible for Ser-385 phosphorylation, we screened consensus kinase recognition motifs in the sequence encompassing Ser-385 using the prediction tool NetPhorest^28^. As depicted in Supplementary Figure S4a, our analysis identified a site of recognition by PAKs. Together with our finding that both Rac1 and Cdc42, but not RhoA, induce the phosphorylation of HACE1 on Ser-385, this *in silico* prediction strongly suggested a role for group-I PAKs in HACE1 phosphorylation. To assess whether group-I PAKs directly target HACE1, we performed *in vitro* kinase assays with recombinant PAK1 and HACE1. As shown in Fig. 3a, we found that HACE1(WT) gets phosphorylated in the presence of PAK1. This revealed that HACE1 is a direct substrate of PAK1. We then analyzed the role of group-I PAK kinases in HUVECs and MCF12A cells. Because PAK3 expression is restricted to the brain^5^, we focused on PAK1, which is notably expressed in mammary glands, and on PAK2, which is ubiquitously expressed. We first verified PAK1 and PAK2 expression in HUVECs and MCF12A cells using specific anti-PAK1 and anti-PAK2 antibodies. This established that PAK2 is expressed both in HUVECs and MCF12A cells, while PAK1 is only expressed in MCF12A cells (Fig. 3b). Using three independent siRNAs specifically targeting PAK2, we found that depletion of PAK2 markedly reduced Rac1(Q61L)-induced phosphorylation of Ser-385 in HUVECs and MCF12A cells (Fig. 3c,d). Additionally, depletion of PAK2 also reduced the CNF1- and Cdc42(Q61L)-mediated phosphorylation of Ser-385 (Supplementary Figure S4b,c). This result suggests that PAK2 acts downstream of Rac1 and Cdc42 to induce HACE1 phosphorylation. Because PAK1 is expressed in MCF12A cells, we assessed its role on HACE1 phosphorylation in these cells and found that silencing of PAK1 reduced Rac1(Q61L)-induced phosphorylation of HACE1 (Fig. 3e). Using an antibody recognizing the phospho-activation sites in the kinase domain of PAK1 and PAK2, we verified that CNF1 treatment or expression of Rac1(Q61L) and Cdc42(Q61L) induced a *bona fide* phospho-activation of PAK1 and/or PAK2 (Supplementary Figure S4d,e). Together, these data establish that PAK1 and PAK2 kinases both regulate the phosphorylation of HACE1 on Ser-385 downstream of Rac1 and Cdc42 activation. Consistently, we found that treatment of MCF12A cells with the group-I PAK inhibitor FRAX597 suppresses HACE1 Ser-385 phosphorylation induced by Rac1(Q61L) and CNF1 (Fig. 3f). Similarly, expression of the dominant negative Kinase Inhibitory Domain of PAK2 (KID2) reduces the levels of Ser-385 phosphorylation induced by Rac1(Q61L) (Fig. 3g). Finally, we found that expression of a constitutively active PAK1(K141A), which is mutated in the auto-inhibited domain, could promote HACE1 phosphorylation on Ser-385 (Fig. 3h). Similar results were obtained with two other constitutively active PAK1 mutants^29,30^ (Supplementary Figure S4f). Thus, PAK1 activation is sufficient to induce HACE1 phosphorylation of Ser-385 in MCF12A cells. Altogether, these results indicate an essential role for group-I PAK kinases in regulating HACE1 phosphorylation at Ser-385 downstream of Rac1 in a variety of cell types.

### Phospho-mimetic mutation S385E hampers HACE1 ability to ubiquitinate Rac1 in cells

**Figure 4:**
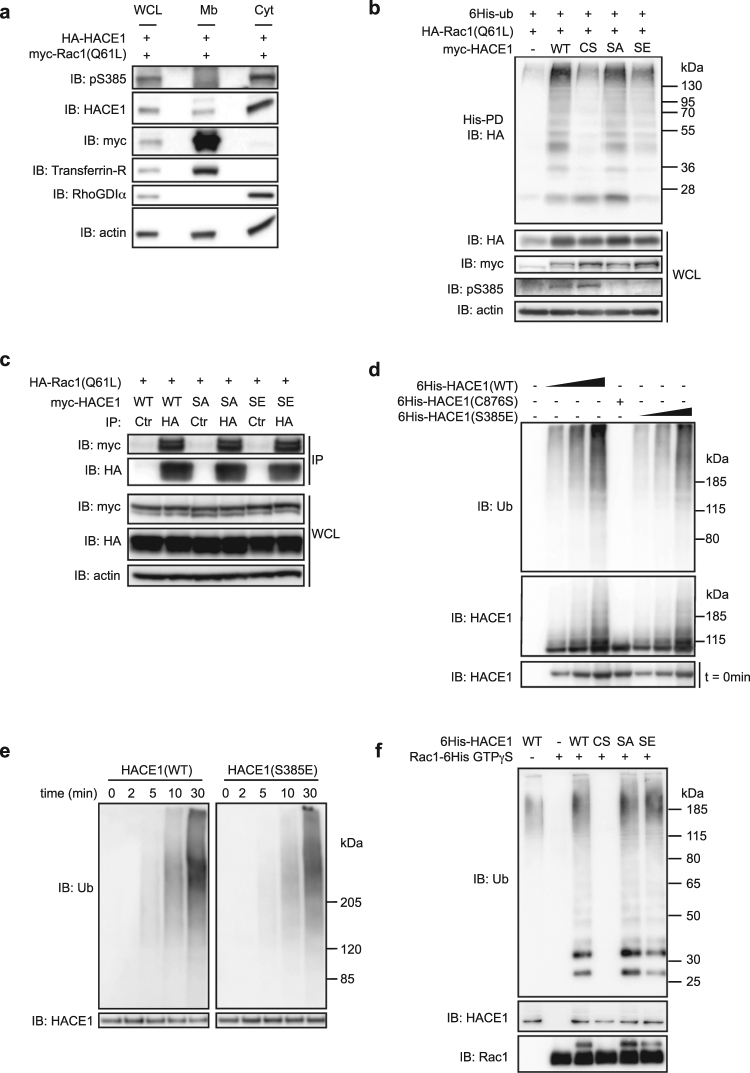
Ser-385 phosphorylation modifies HACE1 properties in cells. (**a**) Cytosol and membrane fractions from MCF12A cells transfected with HA-HACE1 and myc-Rac1(Q61L) were analyzed by immunoblot (IB) using the indicated antibodies. Anti-RhoGDIα and anti-Transferrin-R antibodies are used as specific markers of cytosol and membrane fraction, respectively. (**b**) Protein lysates from MCF12A cells transfected with the indicated plasmids were subjected to His pull-down (His-PD) prior to immunoblot analysis (IB). Whole cell lysate (WCL) IB analysis showing total protein expression. (**c**) Protein lysates from MCF12A cells transfected with the indicated plasmids were subjected to immunoprecipitation (IP) using Ctrl or HA antibodies prior to immunoblot analysis (IB). Whole cell lysate (WCL) IB analysis shows total protein expression. (**d**) *In vitro* ubiquitination assay using recombinant 6His-tagged HACE1(WT), catalytic inactive mutant C876S (CS) and HACE1(S385E) (SE) analyzed 30 min post-reaction by immunoblot using the indicated antibodies. IB at t = 0 min shows the input protein levels. (**e)** *In vitro* ubiquitination assay using HACE1(WT) and HACE1(S385E) (SE) analyzed by IB at the indicated time points. (**f**) *In vitro* ubiquitination assay using recombinant 6His-tagged HACE1(WT), catalytic inactive mutant HACE1(C876S) (CS), HACE1(S385A) (SA), HACE1(S385E) (SE) and Rac1 loaded with GTPγS and analyzed 30 min post-reaction by immunoblot using the indicated antibodies. IBs in (**a–f**) are cropped from the full-length blots shown in Supplemental Figure S12.

We next sought to determine the subcellular localization of Ser-385 phosphorylated HACE1. Using a cell fractionation assay, we found that the phosphorylated form of HACE1 is located in the cytosol while total HACE1 is found both in cytosol and in membrane fractions (Fig. 4a). Since active Rac1 is located at the membrane, Ser-385 phosphorylation likely segregates HACE1 away from its target therefore pointing for an inhibitory effect of this post-translational modification. We went on to determine whether Ser-385 phosphorylation could alter HACE1 capacity to ubiquitinate active Rac1. This question was addressed by conducting an assay based on the purification of cellular proteins cross-linked to 6His-tagged ubiquitin^31^. In accordance with our hypothesis, we found that the phospho-mimetic mutant HACE1(S385E) displays a reduced capacity to ubiquitinate Rac1(Q61L) while the phospho-resistant mutant HACE1(S385A) induces Rac1(Q61L) ubiquitination as efficiently as HACE1(WT) (Fig. 4b). Since the cytosolic phosphorylated form of HACE1 likely segregates away from active Rac1, we explored the impact of Ser-385 phosphorylation on HACE1 association with Rac1(Q61L) by co-immunoprecipitation (co-IP) assay using phospho-mutants of HACE1. We found that HACE1(S385E) as well as HACE1(S385A) interact with Rac1(Q61L) to the same extent as HACE1(WT) (Fig. 4c). In accordance with this result, we found that HACE1(S385E) did not recapitulate the spatial regulation triggered by phosphorylation of Ser-385 as HACE1(S385E) displays both a cytosolic and a membrane localization (data not shown). Thus, the reduced capacity of HACE1(S385E) to ubiquitinate active Rac1 is not due to a reduced association with its target but might instead be explained by a modulation of its enzymatic activity.

We thus next explored whether phosphorylation on Ser-385 down-regulates the intrinsic catalytic activity of HACE1. A classical way to assess the catalytic activity of an E3 ligase is to measure its auto-ubiquitination levels. However, we could not detect the specific auto-ubiquitination of HACE1 (Supplementary Figure S5), indicating that HACE1 does not induce its own ubiquitination in MCF12A cells, which prevented us from using this approach. To overcome this technical obstacle, we performed *in vitro* ubiquitination experiments using purified recombinant 6His-tagged HACE1(WT) and mutants. With this assay, we could detect auto-ubiquitination of HACE1, as evidenced by the ubiquitination of HACE1(WT) and the absence of ubiquitination of the catalytic inactive mutant HACE1(C876S) (Fig. 4d). This indicated that the *in vitro* ubiquitination assay is a reliable method to examine HACE1 catalytic activity. Measuring HACE1 auto-ubiquitination using this assay showed no drastic decrease in HACE1(S385E) activity compared to HACE1(WT). Indeed, we measured a proportional increase in auto-ubiquitination with both forms of HACE1 by using increasing amounts of HACE1(WT) and HACE1(S385E) (Fig. 4d). We also found that HACE1(WT) and HACE1(S385E) display similar auto-ubiquitination kinetics (Fig. 4e). Taken collectively, these results indicate that the phospho-mimetic mutation S385E does not alter the intrinsic catalytic activity of HACE1 *in vitro*. In good agreement with our findings that HACE1(S385E) retains its catalytic activity, we also determined that HACE1(S385E) induces the ubiquitination of recombinant Rac1 loaded with GTPγS *in vitro* (Fig. 4f). Altogether, these results established the paradoxical impact of the S385E mutation on HACE1 activity measured *in vitro* versus *in vivo*. Indeed, we found that HACE1(S385E) is catalytically active on Rac1 *in vitro* (Fig. 4f), though it displays low activity in cells (Fig. 4b), suggesting the combined involvement of Ser-385 phosphorylation and an inhibitory cellular factor that has yet to be identified to down-modulate HACE1 activity.

### Phosphorylation of Ser-385 modulates HACE1 homo-oligomerization

**Figure 5:**
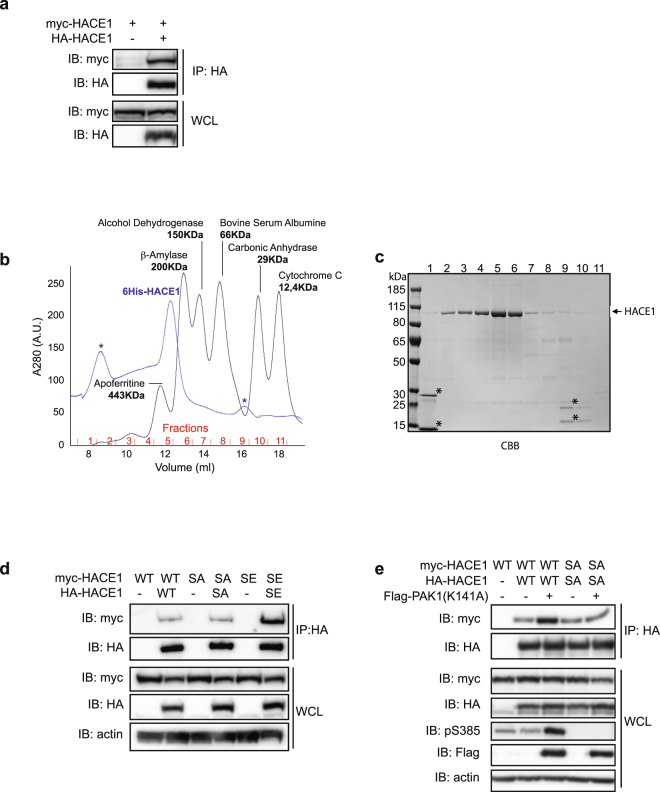
Ser-385 phosphorylation modulates HACE1 oligomerization. (**a**,**d**–**e**) Protein lysates from MCF12A cells transfected with the indicated plasmids were subjected to immunoprecipitation (IP) using HA or Flag antibodies prior to immunoblot analysis (IB). Whole cell lysate (WCL) IB analysis shows the total protein expression. SA is S385A and SE is S385E. (**b)** Superposition of the size exclusion chromatograms from recombinant 6His-HACE1(WT) and the molecular weight markers suggests that HACE1 oligomerizes as a dimer or a trimer. Absorbance at 280 nm is expressed in arbitrary units (A.U.). *Non-specific picks. (**c**) Elution fractions from 6His-HACE1 SEC analyzed by SDS-PAGE and Coomassie Brilliant Blue staining (CBB). *Non-specific contaminants of 6His-HACE1 purification fractions corresponding to the non-specific picks seen in (**b**). IBs in (**a**,**d**,**e**) are cropped from the full-length blots shown in Supplemental Figure S13.

It has previously been shown that several HECT-E3 ligases are regulated by phospho-dependent intra- or inter-molecular interactions^32,33^. To determine whether HACE1 undergoes such regulation in cells, we first performed co-IP experiments using a combination of epitope-tagged HACE1 constructs. We found that HA-HACE1(WT) binds to myc-HACE1(WT) (Fig. 5a), suggesting that HACE1 can form oligomers through inter-molecular interactions. To establish that HACE1 can oligomerize, we next performed size exclusion chromatography (SEC) using recombinant 6His-HACE1 purified from *E*. *coli*. By comparing the elution profiles of 6His-HACE1 with molecular weight markers, we found that 6His-HACE1 is efficiently eluted at an apparent molecular weight of ≥200 KDa (Fig. 5b). With a theoretical molecular weight of monomeric 6His-HACE1 of 106 KDa, this SEC result indicates that HACE1 can directly form homo-oligomers of at least two proteins. To narrow down the HACE1 domains that are engaged in the oligomerization, we performed co-IP experiments using different tagged versions of full length (FL) HACE1 or deletion mutants as depicted in Supplementary Figure S6a. As shown in Supplementary Figure S6b, we confirmed that HACE1 FL binds to HACE1 FL. We also detected an efficient interaction between HACE1 FL and HACE1 ANK + MID (Supplementary Figure S6b). A weaker binding of HACE1 FL with the HECT domain alone was also detected. To confirm these results, we performed the reverse co-IP and found a similar binding of HACE1 ANK + MID with HACE1 FL. In these conditions, we also detected a strong interaction between HACE1 ANK + MID with the HECT domain alone (Supplementary Figure S6c,d). Altogether, these results indicate that HACE1 can form homo-oligomers that most likely involve interactions between the ANK + MID region and the HECT domain.

We next sought to determine whether phosphorylation of Ser-385 can interfere with HACE1 homo-oligomerization. We found that HA-HACE1 binds to myc-HACE1 to the same extent as HA-HACE1(S385A) with myc-HACE1(S385A) (Fig. 5d). This suggests that HACE1 homo-oligomerization occurs in the absence of Ser-385 phosphorylation. Interestingly, we found that HACE1(S385E) displays higher inter-molecular interaction levels than HACE1(WT) or HACE1(S385A), suggesting that phosphorylation modifies the state of HACE1 homo-oligomerization (Fig. 5d). In accordance with this result, we found that overexpression of active PAK1(K141A) greatly increases HACE1 inter-molecular interaction levels (Fig. 5e). This is specific to Ser-385 phosphorylation as PAK1(K141A) overexpression did not modify the extent of HACE1(S385A) inter-molecular interactions (Fig. 5e). Altogether, these results indicate that the phosphorylation at Ser-385 modulates HACE1 homo-oligomerization properties.

## Discussion

Using an unbiased proteomics approach, we have identified HACE1 Ser-385 as a pivotal amino acid residue that is phosphorylated in response to CNF1 or VEGF treatments. We have shown that expression of activated Rac1 or Cdc42 is sufficient to induce HACE1 phosphorylation and that Rac1 plays a major role in the phosphorylation of HACE1 downstream CNF1. Our data point to group-I PAKs as being responsible for the phosphorylation of HACE1 downstream of Rac1. Indeed, we found that inhibition of PAK1 or PAK2 is sufficient to block Rac1- or Cdc42-induced phosphorylation of Ser-385. In addition, we demonstrated that PAK1 phosphorylates directly HACE1 *in vitro*. Finally, we report that the phosphorylation of Ser-385 is critical for the modulation of the capacity of HACE1 to ubiquitinate Rac1 as well as its oligomerization state. Our data support a model where Rac1 down-regulates the activity of its own E3 ubiquitin ligase likely as a feedback mechanism. Future studies using genome editing approaches will help to determine the signaling impact of the Ser-385 phosphorylation of HACE1 on endogenous Rac1 stability and activity as well as its consequences on cellular homeostasis.

How can phosphorylation of Ser-385 reduce the capacity of HACE1 to ubiquitinate Rac1? Phosphorylation sites can serve as recognition sites for adaptor proteins such as the 14-3-3 family of proteins that modify localization of the phosphorylated proteins and/or keep them away from their protein partners^34^. For instance, phosphorylation of the E3-HECT Nedd4-2 has been shown to trigger its association with cytosolic 14-3-3 proteins, which prevents Nedd4-2 from binding to its transmembrane target — the epithelial Na^+^ channel (ENaC)^35^. Here, we have observed that the phosphorylated form of HACE1 is mainly located in the cytosol while total HACE1 is present in both in the cytosol and at the membrane (Fig. 4a). This cytosolic localization of phosphorylated HACE1 seems to be independent of 14-3-3 proteins as we failed to detect a specific association between the Ser-385-phosphorylated form of HACE1 and 14-3-3 proteins (Supplementary Figure S7). It is conceivable that phosphorylation of Ser-385 reduces the pool of active HACE1 at the membrane where Rac1 ubiquitination by HACE1 occurs^3^. We found that HACE1(S385E) localized both in membranes and cytosol (data not shown) indicating that an additional mechanism might confer inhibitory properties to the phospho-mimetic mutant.

To determine how the phospho-mimetic mutant HACE1(S385E) has a reduced capacity to ubiquitinate Rac1(Q61L), we have investigated whether the phosphorylation of HACE1 might directly affect its binding to active Rac1. Indeed, this is the case for the HECT E3 ligase Smurf1 whose phosphorylation modulates its own affinity for its substrates Par6 and RhoA^36^. However, we found that the HACE1(S385E) phospho-mimetic mutant binds to Rac1 as efficiently as HACE1(WT). Therefore, we hypothesized that the phosphorylation of Ser-385 might regulate HACE1 catalytic activity. Indeed, such a regulation has been previously reported for Itch/AIP4 and Nedd4-1, whose activities are regulated upon JNK- and Src-dependent phosphorylation, respectively^32,33^. However, the absence of HACE1 auto-ubiquitination activity in our cellular model precluded us from testing this possibility. Nevertheless, *in vitro* ubiquitination experiments indicate that HACE1(S385E) is functional, as it can ubiquitinate itself and Rac1. This important result allowed us to discard the possibility that the lowered HACE1(S385E) activity measured in cells is due to intrinsic alterations in the protein. The paradoxical differences between HACE1(S385E) activities *in vitro* and *in vivo* suggest that, in addition to Ser-385 phosphorylation, a cellular adaptor mediates HACE1 inhibition. Such regulation requiring both the phosphorylation of the HECT E3 ligase and an adaptor protein has been recently described for HUWE1 (aka MULE, ARF-BP1)^37^. Indeed, although it works in the opposite direction, it was shown that phosphorylation of HUWE1 disrupts the interaction with its inhibitory co-factor p14-ARF and ultimately activates the E3 ligase^37^. A similar scenario involving the phospho-dependent binding of a regulatory adaptor to HACE1 is likely to occur here. One could hypothesize that an activator can bind the non-phosphorylated form of HACE1 to trigger its activity or, conversely, that an inhibitor binds the phospho-form of HACE1 to abrogate its activity. Therefore, our work opens new avenues to search for regulatory adaptors whose binding is modulated by HACE1 phosphorylation at Ser-385.

In most cases, the regulation of HECT E3 ligases involving phosphorylation and/or adaptors is the consequence of structural modifications. This has been particularly well-described in the Nedd4 family, which undergoes inhibitory intra- or intermolecular interactions between their C-terminal HECT domain and C2 or WW N-terminal domains^38,39^. These interactions, whether they induce a closed monomeric conformation or the formation of an inactive homodimer, are relieved by phosphorylation or binding to an adaptor protein^32,33,40–44^. Similarly, it has recently been proposed that the HUWE1 E3 ligase can adopt an inhibitory asymmetric homodimeric conformation that leads to its inhibition and that binding of p14-ARF shifts the dynamic conformational equilibrium of HUWE1 toward the inhibitory dimer^45^. Our co-IP data indicate that HACE1 forms inter-molecular interactions that involve the binding of the HECT domain to the ANK + MID region. Interestingly, the HECT domain does not bind to either the ANK domain or the MID region alone. This suggests that either the HECT domain binds a motif intersecting the ANK and MID regions or that the ANK + MID region adopts a particular conformation that enables its binding to the HECT domain. In line with this second hypothesis, we have recently demonstrated that the cooperation of ANK and MID regions is important for the selective binding of HACE1 to the active form of Rac1^23^. Here, we found that recombinant HACE1 can form a homo-oligomer *in vitro* by SEC analysis. This indicates that the HACE1 inter-molecular interactions found in cells are likely direct. Moreover, our SEC data also indicates that HACE1 homo-oligomers correspond to dimers or trimers. The formation of trimers of HECT E3 ligases has been reported for Ube3A/E6AP, which promotes full activity of the ligase^46^. Interestingly, an independent study has shown that phosphorylation of threonine 485 inhibits Ube3A/E6AP catalytic activity^47^. Whether this phosphorylation alters Ube3A/E6AP trimerization has not been explored. However, mutation of this specific threonine is associated with autism, demonstrating the importance of this phospho-regulation in physiology. A recent study has shed light on the importance of post-translational modifications in the trimerization of several Nedd4 proteins^48^. It was shown that the formation of Nedd4 trimers is promoted by ubiquitination of their HECT domains, which leads to their inactivation^48^. Our data indicate that homo-dimerization or homo-trimerization of HACE1 occurs in the absence of Ser-385 phosphorylation as the phospho-resistant mutant HACE1(S385A) and HACE1(WT) display similar levels of inter-molecular interactions. Interestingly, our results suggest that phosphorylation of Ser-385 modifies HACE1 oligomerization properties. Indeed, we found higher levels of HACE1(S385E) inter-molecular interactions compared with HACE1(WT). Similarly, specific phosphorylation of Ser-385 induced by PAK1(K141A) increases HACE1(WT) inter-molecular interactions. This increase of interactions may thus reflect an increase in the stability of HACE1 oligomer or an increase in the extent of oligomerization, i.e., number of units per oligomer. Moreover, our data indicate that an adaptor is likely required to account for the decrease in HACE1(S385E) activity in cells. Although it remains to be strictly demonstrated that HACE1 phospho-oligomerization plays a direct inhibitory role on its activity, one could hypothesize that an adaptor binds to the phospho-oligomer to mediate HACE1 inhibition or that an adaptor binds to HACE1 once it is phosphorylated on Ser-385, which in turn modifies the properties of HACE1 to oligomerize into an inactive complex. Interestingly, we found that Ser-385 is located within an intrinsically disordered region (IDR)^49^ (Supplementary Figure S8**)**. These IDRs, which lack stable tertiary structures, are phosphorylation hotspots^50^; thanks to their flexibility, IDRs allow easy access and recognition of their phosphorylated residue to binding surfaces^51^. Further work remains to be completed to determine how phosphorylation of Ser-385 modifies HACE1 oligomerization properties. In conclusion, we have uncovered a pivotal role for Ser-385 in the regulation of HACE1 that sets the basis for deciphering the relationship between the structure and activity of HACE1.

## Methods

### Plasmid constructs, primers, siRNAs, antibodies and reagents

All plasmids used in this study are listed in Supplementary Table S1. Site-directed mutagenesis were performed using the primers listed in Supplementary Table S2 and the QuickChange Lighting (Agilent) and QuickChange II (Agilent) kits for HACE1 and PAK1 plasmids, respectively. The pSY5M-6His-PAK1 plasmid was obtained by subcloning the rat PAK1 sequence^52^ into the pET21d + vector. pXJ-GST-KID2 was obtained by subcloning the rat PAK2 kinase inhibitory domain (85-144 aa) into the pXJ40-GST vector using the XhoI/KpnI restriction sites. All the plasmid sequences were verified by sequencing. SMART-pool siRNA mixes against PAK1 (#5058) and PAK2 (#5062) were acquired from GE Dharmacon. The silencer select siRNAs against PAK2 were labeled as siPAK2#1 (s10022), siPAK2#2 (s10023), and siPAK2#3 (s10024) and were purchased from Ambion® ThermoFisher Scientific. All the antibodies used in this study are listed in Supplementary Table 3. The rabbit anti-phospho-HACE1S385 (pS385) and anti-phospho-HACE1S337 (pS337) antibodies were raised against the phospho-peptide sequences KNKRD[pS]TEITS and FRIGPS(pS)PSNGID and purified by positive and negative affinity purification using the phosphorylated and unphosphorylated peptide sequences, respectively (Perbio Science France SAS). The CNF1 toxin was purified as described in Doye *et al*., 2006^53^. The PAK1 kinase inhibitor FRAX597 (Selleckchem) was used at 2 or 5 µM for 16 hours and the mTOR inhibitors Torin1 (Tocris) and Rapamycin (Sigma-Aldrich) were used at 0.1 µM and 0.1 nM respectively, for 4 hours. VEGF (Peprotech) was used at 20ng/ml for 10 min.

### Cell culture, transfection and lysis

HUVECs were obtained from PromoCell and maintained in human endothelial SFM medium (Gibco^TM^) supplemented with 20% fetal bovine serum (Gibco^TM^), 20 ng/ml FGF-2, 10 ng/ml EGF (Peprotech), 1 µg/ml heparin (Sigma-Aldrich), and 1% penicillin-streptomycin (Gibco^TM^). MCF-12A mammary gland epithelial cells (CRL-10782, ATCC) were maintained in DMEM/F12 (Gibco^TM^) supplemented with 7.5% horse serum (Biowest), 20 ng/ml recombinant human EGF (Peprotech), 10 µg/ml human recombinant insulin (Gibco^TM^), 0.5 μg/ml hydrocortisone (Sigma-Aldrich), 100 ng/ml cholera toxin (Sigma-Aldrich), and 1% penicillin-streptomycin (Gibco^TM^). Human embryonic kidney (HEK293) cells were maintained in DMEM (Gibco^TM^) supplemented with 10% fetal bovine serum and 50 µg/ml gentamicin (Gibco^TM^).

Plasmid DNAs and siRNAs were transfected into MCF12A cells using Lipofectamine LTX and Lipofectamine RNAiMAX, respectively, according to manufacturer’s procedures (Invitrogen). For DNA transfection, cells were seeded at 70% confluence 16–24 hours before DNA transfection. For DNA and siRNA co-transfection, 1.3 × 10^6^ cells were directly reverse transfected with a 150 nM final concentration of siRNA in a 12-well plate, incubated for 48 hours, transfected with plasmid DNA and incubated an additional 24 hours before lysis. HUVECs were transfected with plasmid DNA by electroporation or using the PolyMag reagent (OZ Biosciences) as described previously^53^ 24 hours before lysis unless otherwise stated. HUVECs were transfected with siRNAs using the PolyMag reagent at a final concentration of 50 mM and incubated for 72 hours before lysis. For DNA and siRNA co-transfection, cells were first transfected with siRNA using PolyMag, incubated for 48 hours, transfected with plasmid DNA using PolyMag and incubated an additional 24 hours before lysis. HEK293 cells were transfected with Lipofectamine 2000 according to manufacturer’s procedure (Invitrogen).

For the analysis of the total protein levels by immunoblot, cells were lysed in 1× RIPA buffer (Bio Basic) supplemented with protease and phosphatase inhibitors (Pierce) and the lysates were analyzed immunoblot.

For cytosol – membrane fractionation, cells were lysed in SI buffer (Sucrose 250 mM, Imidazole 3 mM) and centrifuged at 10.000 g 4 °C for 10 min to precipitate the nucleus. The supernatant was recovered, part (10%) was kept for a post-nuclear “input” control and the rest was centrifuged at 100.000 g 4 °C for 1 hour. The supernatant corresponds to the cytosolic fraction and the pellet to the membrane (and organelles) fraction. Both fractions and the input control were analyzed by immunoblot.

### Immunoblot analysis

Protein lysates were resolved using NuPAGE 3–8% Tris Acetate, 4–12% Bis-Tris pre-cast gels (Invitrogen) or 8%, 10% or 12% Tris Glycine SDS-PAGE gels. Separated proteins were transferred onto Immobilon-PVDF membranes (Millipore) using a semi-dry method (Trans-blot® Turbo™ transfer, Biorad) or by overnight transfer in carbonate buffer (1.25 mM NaHCO_3_, 0.37 mM Na_2_CO_3_, and 20% v/v ethanol, pH 9.9). Membranes were probed with the indicated primary and secondary antibodies (Supplementary Table S3), and then incubated with the Immobilon Western Chemiluminescent HRP substrate (Millipore). The emitted chemiluminescent signals were detected with a Syngene Pxi4 imaging system (ThermoFisher Scientific). When appropriate, immunoblot signals were quantified by densitometry using the Image Studio 3.1.4 software.

### Tandem mass spectrometry analysis

HUVECs transfected with HA-HACE1 expressing plasmid were left untreated or were treated with CNF1 at 10^−9^ M for 24 hours. Following lysis and an immunoprecipitation assay, immuno-purified HA-HACE1 from both conditions were separated on an SDS–PAGE gel, and trypsin-digested samples obtained from the HA-HACE1 cut gel slices were analyzed as described previously^54^. Briefly, proteins were digested in-gel using trypsin (Gold, Promega). The generated peptides were analyzed online using an LTQ Orbitrap Elite mass spectrometer (Thermo Fisher Scientific) coupled to an Ultimate 3000 HPLC (Dionex, Thermo Fisher Scientific). Desalting and pre-concentration of the samples was performed online on a Pepmap^®^ pre-column (0.3 mm × 10 mm, Dionex). A gradient consisting of 0–40% B in A for 60 min, followed by 80% B and 20% A for 15 min (A was 0.1% formic acid with 2% acetonitrile in water and B was 0.1% formic acid in acetonitrile) at 300 nl/min was used to elute peptides from the capillary reverse-phase column (0.075 mm × 150 mm, Pepmap^®^, Dionex). Eluted peptides were electrosprayed online at a voltage of 1.9 kV into an LTQ Orbitrap Elite mass spectrometer. A single full-scan mass spectrum cycle (400–2000 *m*/*z*) at a resolution of 120,000 (at 400 *m*/*z*) in the orbitrap, followed by twenty data-dependent MS/MS spectra were repeated continuously throughout the nanoLC separation. All the MS/MS spectra (acquired using the linear trap quadrupole) were recorded using a normalized collision energy (33%, activation Q of 0.25 and activation time of 10 ms).

Spectral data were analyzed using the MaxQuant software^55^ with standard settings and the following variable modifications: (1) Acetyl (Protein N-term), (2) Oxidation (M), and (3) Phosphorylation (STY). For quantification, signal extraction of the identified peptides was performed using Skyline^56^.

### Co-Immunoprecipitation and His-Ub pull-down

For co-immunoprecipitation, MCF12A cells were scraped 20 hours post-transfection in 1 ml of immunoprecipitation buffer (SLB) (50 mM Tris-HCl, pH 7.5, 150 mM NaCl, 1% (v/v) Triton-X100, and 0.27 M sucrose) supplemented with protease and phosphatase inhibitors (Pierce) and lysed by a freeze-thaw cycle in liquid nitrogen. The cleared lysates were incubated with 30 μl of Ezview Red Anti-HA or anti-Flag Affinity gel (Sigma-Aldrich) for 2 hours at 4 °C. Beads were washed at least twice with 1 ml of SLB and resuspended in 30 μl of 1× LDS buffer with 50 mM DTT. For the His-Ub pull-down experiments, MCF12A cells were lysed 7 hours post-transfection in ULB (8 M Urea, 20 mM Tris-HCl [pH 7.5], 200 mM NaCl, 10 mM imidazole, and 0.1% Triton X-100). The proteins that covalently bound to 6His-tagged ubiquitin were pulled-down by incubating 95% of the cleared lysate with 30 µl cobalt-chelated resin (Clontech), which was previously blocked in ULB + 5% bovine serum albumin (RIA grade, Sigma) for 1 hour. After lysate incubation, the beads were washed four times in ULB and resuspended in one volume of Laemmli’s buffer.

### *In vitro* kinase assays

Purified PAK1 (2 ng/µl) was incubated with recombinant purified HACE1 (40 ng/µl) and 30 µM ATP (10 μCi of [γ 32 P]-ATP) in kinase buffer (25 mM HEPES pH 7.3, 0.02% Triton X-100, 25 mM NaCl, 5 mM β-glycerophosphate, 2.5 mM NaF, 5 mM MgCl_2_, and 0.1 mM MnCl_2_) at 30 °C for 30 min in a final volume of 50 µl. Samples were analyzed by immunoblot followed by autoradiography and Coomassie blue staining.

### Purification of recombinant proteins by IMAC

*E*. *coli* BL21 strains transformed with pET-28a plasmids encoding 6His-HACE1 (WT) and mutants were grown for 24 hours at 37 °C in 1 L of Luria-Bertani (LB) Broth with kanamycin 50 µg/ml. Isopropyl β-D-1-thiogalactopyranoside (IPTG) was added at a final concentration of 100 µM and the culture was grown for another 8 hours at 30 °C. Bacteria were harvested by centrifugation and lysed using a French Press in 20 ml of Buffer A (Tris-HCl pH 7.5 and 200 mM NaCl) with 1 mM phenylmethylsulfonyl fluoride (PMSF). The cleared lysate was subjected to Immobilized Metal Affinity Chromatography (IMAC) using a Chelating Sepharose Fast Flow column (GE healthcare Life Science) charged with NiSO_4_ in an AKTA system with the UNICORN software (GE healthcare Life Science). Elution was performed with Buffer A containing increasing concentrations of imidazole. The approximately 15 ml fraction eluted with 250 mM imidazole that contained most of the 6His-HACE1 was dialyzed overnight in 25 mM Tris-HCl with 125 mM NaCl and concentrated the next morning to 1 µg/µl using an Amicon Ultra-15 50 K (Millipore).

*E*. *coli* BL21 strains transformed with pSY5M plasmids encoding 6His-PAK1 were grown overnight at 30 °C in 50 ml of LB with 50 µg/ml chloramphenicol and 50 µg/ml ampicillin and incubated overnight (ON) at 30 °C. The next morning, 200 ml of LB with antibiotics was inoculated with 20 ml of the overnight culture. When 0.6 < OD600 < 1, IPTG was added to a final concentration of 500 µM and the culture was grown another 4 hours at room temperature with shaking. The bacteria were harvested by centrifugation and lysed by sonication in 10 ml of cold bacterial lysis buffer (50 mM Tris [pH 8.0], 0.5% Triton-X100, 5 mM MgCl_2_, 20 mM imidazole, 1 mg/ml lysozyme, 5 mM DTT, 0.5 mM PMSF and 1× Protease Inhibitors Cocktail (Roche)). The cleared lysate was incubated with 250 µl of Ni-NTA-agarose slurry and roll at 4 °C for 2 hours. The 6His-PAK1 bound to the Ni-NTA-agarose beads was transferred into a 10-ml disposable column, washed and eluted in 5 × 1 ml of elution buffer (50 mM Tris [pH 8.0], 0.5% Triton-X100, 5 mM MgCl_2_, 250 mM imidazole and 5% glycerol). Fractions with >1 mg/ml of 6His-PAK1 were pooled, aliquoted and snap-frozen in liquid nitrogen.

### Size-exclusion chromatography

Size Exclusion Chromatography (SEC) was performed using 500 µl of IMAC-purified 6His-HACE1 (WT) at 1 µg/µl using a Superdex 200 Increase 10/300 GL with an AKTA system and the UNICORN software program (GE Healthcare Life Sciences). A mix of *gel filtration marker kit for protein molecular weights 12*,*000–200*,*000* *Da* (Sigma-Aldrich) with *apoferritin from equine spleen* (Sigma-Aldrich) was used as the control.

### *In vitro* ubiquitination assay

HACE1 auto-ubiquitination and HACE1-induced ubiquitination of Rac1 reactions were performed in a 40 µl final volume containing 20 mM Tris-HCl [pH 7.5], 10 mM MgCl_2_, 5 mM ATP, and 1 mM DTT using 250 ng of recombinant human 6His-Ube1 (RD system), 500 ng of recombinant human UbcH7 (RD system), 1 µg of ubiquitin (RD system), 100 ng to 1 µg of 6His-HACE1 and 1 µg of Rac1–6His that was previously loaded with GTP-γS. Reactions were incubated at 37 °C for 30 min unless otherwise indicated and were stopped by the addition of 1× LDS and 1× reducing agent (ThermoFisher Scientific).

### Lambda phosphatase treatment

One thousand units of λ-Protein Phosphatase (Sigma-Aldrich) were used to dephosphorylate 50 µg of proteins from whole cell lysates. Samples were incubated for 40 min at 30 °C, and the reactions were stopped by the addition of 1× LDS and 1× Reducing Agent (ThermoFisher Scientific).

### Bioinformatics analysis

Prediction of IDRs was performed using the online tool PONDR predictor with the VL-XL, XL1-XT and CaN-XT algorithms http://www.pondr.com^49^. The PAK consensus kinase sequence was obtained using the online tool NetPhorest http://www.netphorest.info/index.shtml^28^.

### Statistical analysis

The data were analyzed with the statistical software Graphpad Prism 6.0 f. Statistical significance was evaluated by one- or two-sample unpaired t-tests.

## Electronic supplementary material

Supplementary Information
