# Supplementary material for: Group-I PAKs-mediated phosphorylation of HACE1 at serine 385 regulates its oligomerization state and Rac1 ubiquitination
Source: Sci Rep. 2018 Jan 23;8:1410. doi: 10.1038/s41598-018-19471-2 (PMC5780496; doi:10.1038/s41598-018-19471-2)

Supplementary Information

Group-I PAKs-mediated phosphorylation of HACE1 at serine 385 regulates its oligomerization state and Rac1 ubiquitination

Maria I. Acosta, Serge Urbach, Anne Doye, Yuen-Wai Ng, Jérôme Boudeau, Amel Mettouchi, Anne Debant, Edward Manser, Orane Visvikis, Emmanuel Lemichez

Supplementary Figure S1: Identification of two phosphorylation sites of HACE1.

a-d. Fragmentation spectra of HACE1 phospho-peptides showing that Ser-337 and Ser-385 are phosphorylated. **e.** Graph showing the occurrence of HACE1 phospho-peptides in CNF1 treated cells relative to control conditions. **f.** Protein lysates from HUVECs transfected with HA-HACE1(WT) and treated with CNF1 at 10^{-9} M for 24 hours were treated or not with λ-phosphatase (λ-PPase) and analyzed by immunoblot (IB) using the indicated antibodies. IB: actin is used as a loading control. **g.** Graph showing levels of pS337 relative to HACE1 total protein levels quantified by densitometry from the IB analysis. **e-g**. Data correspond to the mean ± SD of > 3 biological replicates. p value was determined by one-sample t-test.

Supplementary Figure S2: CNF1 and Rac1 induce phosphorylation at Ser-385.

a-b. Protein lysates from MCF12A cells transfected with HA-HACE1(WT) and (**a**) treated with CNF1 for 24 hours or (**b**) co-transfected with myc-Rac1(Q61L) and analyzed by immunoblot (IB) using the indicated antibodies. IB: actin is used as a loading control.

Supplementary Figure S3: Phosphorylation of HACE1 at Ser-385 is independent of mTOR signaling.

HUVECs transfected with the indicated plasmids were treated with rapamycin (0.1 µM) or Torin1 (0.1 M) for 4 hours prior to lysis and analysis by immunoblot (IB) using the indicated antibodies. IB: actin is used as a loading control and pS6 is used as a control for mTOR activation.

Supplementary Figure S4: Group I PAKs induce the direct phosphorylation of HACE1.

a. NetPhorest software analysis of the sequence surrounding Ser-385 identified a conserved PAK consensus motif. **b-c.** Protein lysates from MCF12A cells transfected with siRNA mix targeting PAK2 (**c**) and with plasmids expressing HA-HACE1 and Cdc42(Q61L) or (**b**) intoxicated with CNF1 for 24 hours were analyzed by immunoblot (IB) using the indicated

antibodies. **d-e.** Protein lysates from MCF12A cells transfected with plasmids expressing HA-HACE1 and (**d**) Rac1(Q61L) or (**e**) Cdc42(Q61L) or (**d**) intoxicated with CNF1 for 24 hours were analyzed by immunoblot (IB) using the indicated antibodies. **f.** Protein lysates from MCF12A cells transfected with HA-HACE1, myc-Rac1(Q61L) and myc-PAK1T423E or myc-PAK1L107F and analyzed by IB using the indicated antibodies.

Supplementary Figure S5: HACE1 does not induce its own ubiquitination in MCF12A cells.

Protein lysates from MCF12A cells transfected with the indicated plasmids were subjected to His pull-down (His-PD) prior to immunoblot analysis (IB). Whole cell lysate (WCL) IB analysis showing total protein expression. CS is C876S, SA is S385A, and SE is S385E.

Supplementary Figure S6: *In vivo* inter-molecular interactions of HACE1 requires HECT binding to the ANK+MID region.

a. Schematic representation of the HACE1 protein indicating the position and size of the ANK and HECT domains as well as the MID region. The structures of the deletion mutants are also represented. **b-d**. Protein lysates from (**b**) HEK293 or (**c-d**) MCF12A cells transfected with the indicated plasmids were subjected to immunoprecipitation (IP) using myc or Flag antibodies prior to immunoblot analysis (IB). Whole cell lysate (WCL) IB analysis shows the total protein expression.

Supplementary Figure S7: Phosphorylation at Ser-385 does not promote a specific association between HACE1 and 14-3-3 proteins.

Protein lysates from MCF12A cells transfected with the indicated plasmids were subjected to immunoprecipitation (IP) using HA antibodies prior to IB. Whole cell lysate (WCL) IB analysis shows the total protein expression.

Supplementary Figure S8: Ser-385 is located within an intrinsically disordered region.

a. Schematic representation of the HACE1 protein that shows Ser-385 within the MID region. **b-g.** HACE IDR prediction using the PONDR® predictor with the (**b-c**) VT-XL, (**d-e**) XL1-XT and (**f-g**) CaN-XT algorithms. (**b, d,** and **f)** Graphs represent the PONDR score relative to the residue position. Scores under and above 0.5 correspond to ordered and disordered regions, respectively. (**c, e,** and **g)** Tables listing the IDR domains found by the 3 algorithms.

Supplementary Figures S9-S13: Full-length blots and gels showed in the main figures.

A dashed box within each full-length blot or gel shows the portion of the image presented in the indicated main figure. The text beside each image indicates the type of sample (IP: immuno-precipitate, WCL: whole cell lysate, His-PD: Histidine-pull down), staining (CBB: coomassie brilliant blue), analysis (Autoradiography, IB: immuno-blot), and the antibody used to probe the membrane in the case of IBs.

Supplementary table S1: Plasmid List

#	Plasmid	Reference	Design
1	PKH3-HA3-HACE1(WT)	Andrio et al. 2017	
2	PKH3-HA3-HACE1(S385A)	This study	mutagenesis of plasmid #1 using primers #1-2
3	PKH3-HA3-HACE1(S385E)	This study	mutagenesis of plasmid #1 using primers #3-4
4	PKH3-HA3-HACE1(C876S)	Torrino et al., 2017	
5	pXJ40-HA-Rac1(Q61L)	Doye et al., 2002	
6	pKH3-HA3-HA-Cdc42(Q61L)	Doye et la., 2006	
7	pKH3-HA3-HA-RhoA(L63)	Doye et la., 2006	
8	pRK5-myc-HACE1(WT)	Andrio et al., 2017	
9	pRK5-myc-HACE1(S385A)	This study	mutagenesis of plasmid #8 using primers #1-2
10	pRK5-myc-HACE1(S385E)	This study	mutagenesis of plasmid #8 using primers #3-4
11	pXJ40-HA -Rac1(T17N)	Doye et el., 2002	
12	pKH3-HA3-Cdc42(T17N)	This study	mutagenesis of pKH3-HA3-Cdc42WT (Doye et al., 2006) using primers #7-6
13	pRK5-myc-Rac1(Q61L)	Visvikis et al., 2008	
14	6His-HACE1(WT)	Torrino et al., 2011	
15	6His-HACE1(C876S)	This study	mutagenesis of plasmid #14 using primers #5-6
16	6His-HACE1(S385A)	This study	mutagenesis of plasmid #14 using primers #1-2
17	6His-HACE1(S385E)	This study	mutagenesis of plasmid #14 using primers #3-4
18	pSY5M-6His-PAK1	This study	See methods
19	pXJ-Flag-PAK1(K141A)	This study	Mutagenesis of plasmid pXJ-Flag-PAK1 (Ng et al., 2010) using primers #7-8
20	pXJ40-GST-KID2 (85-144)	This study	See methods
21	pCMV6M-myc-PAK1(T423E)	Sells et al., 1997	pCMV6M-PAK1 T423E was a gift from Jonathan Chernoff (Addgene plasmid # 12208)
22	pCMV6M-myc-PAK1(L107F)	Xiao et al., 2002	pCMV6M-PAK1 L107F was a gift from Jonathan Chernoff (Addgene plasmid # 12212)
23	pRGB4-6His-Ub	Doye et al., 2006	
24	pKH3-HA3-ANK (1-257aa)	Andrio et al, 2017	
25	pKH3-HA3-MID (258-571aa)	Andrio et al, 2017	
26	pKH3-HA3-HECT (574-909aa)	Andrio et al, 2017	
27	pKH3-HA3-ANK+MID (1-571)	Andrio et al, 2017	
28	pKH3-HA3-MID+HECT (258-909aa)	Andrio et al, 2017	
29	pCMV-Tag2B-Flag-ANK+MID (1-574a)	Andrio et al, 2017	

- Andrio, E. et al. Identification of cancer-associated missense mutations in hace1 that impair cell growth control and Rac1 ubiquitylation. Sci. Rep. 1–11 (2017).

- Doye, A., Boyer, L., Mettouchi, A. & Lemichez, E. Ubiquitin-mediated proteasomal degradation of Rho proteins by the CNF1 toxin. Meth. Enzymol. 406, 447–456 (2006).

- Torrino, S. et al. The E3 ubiquitin-ligase HACE1 catalyzes the ubiquitylation of active Rac1. Dev Cell 21, 959–965 (2011).

- Visvikis, O. et al. Activated Rac1, but not the tumorigenic variant Rac1b, is ubiquitinated on Lys 147 through a JNK-regulated process. FEBS J. 275, 386–396 (2008).

- Ng, Y. W. et al. Why an A-loop phospho-mimetic fails to activate PAK1: understanding an inaccessible kinase state by molecular dynamics simulations. Structure 18, 879–890 (2010).

- Sells, M.A. et al. Human p21-activated kinase (Pak1) regulates actin organization in mammalian cells. J. Curr Biol. 7, 202-210 (1997)

- Xiao, G.H. et al. p21-activated kinase links Rac/Cdc42 signaling to merlin. J Biol Chem. 277, 883-886 (2002).

Supplementary table S2: Primer List

#	Primer name	Sequence
**1**	HACE1(S385A)-Fw	5'- GAA TTG ATG AAA AAC AAA AGA GAC GCA ACA GAG ATC ACT TCT ATT TTA C
**2**	HACE1(S385A)-Rev	5'- GTA AAA TAG AAG TGA TCT CTG TTG CGT CTC TTT TGT TTT TCA TCA ATT C
**3**	HACE1(S385E)-Fw	5'- CAC AGA ATT GAT GAA AAA CAA AAG AGA CGA AAC AGA GAT CAC TTC TAT TTT ACT GAA A
**4**	HACE1(S385E)-Rev	5'- TTT CAG TAA AAT AGA AGT GAT CTC TGT TTC GTC TCT TTT GTT TTT CAT CAA TTC TGT G
**5**	HACE1(C876S)-Fw	5'- CTT TTA CCA ACT TCA AGC ACA TCC ATC AAC ATG CTC AAG
**6**	HACE1(C876S)-Rev	5'- CTT GAG CAT GTT GAT GGA TGT GCT TGA AGT TGG TAA AAG
**7**	Cdc42(T17N)-Fw	5'- GGT GCT GTT GGT AAA AAC TGT CTC CTG ATA TCC TAC
**8**	Cdc42(T17N)-Rev	5'- GTA GGA TAT CAG GAG ACA GTT TTT ACC AAC AGC ACC
**9**	PAK1(K141A)-Fw	5'- GTC AGG CGT ACA TGA GTT TTA C
**10**	PAK1(K141A)-Rev	5'- GTA CGC CTG ACT ATT GGA G

Supplementary table S3: Antibody List

Antibody	Clone Number	Supplier
Mouse monoclonal anti-HA.11	16B12	Biolegend
Rat monoclonal anti-HA	3F10	Roche
Mouse monoclonal anti-GST	26H1	CST
Mouse monoclonal anti-Flag	M2	Sigma-Aldrich
Mouse monoclonal anti-c-Myc	9E10	Biolegend
Rabbit monoclonal anti-HACE1	EPR7962	Abcam
Mouse monoclonal anti β-Actin	AC-74	Sigma-Aldrich
Rabbit polyclonal anti-PAK1	N/A	CST
Rabbit polyclonal anti-PAK2	N/A	CST
Rabbit polyclonal anti-RhoGDIα	N/A	Santa Cruz
Mouse monoclonal anti-Transferrin Receptor	H68.4	ThermoFisher Scientific
Rabbit polyclonal anti-14-3-3 (pan)	N/A	CST
Rabbit polyclonal anti-phospho-PAK1(T423)/PAK(T402)	N/A	CST
Rabbit monoclonal anti-phospho-VEGFR2(T1175)	19A10	CST
Rabbit polyclonal anti-phospho-S6(S235/236)	N/A	CST
Rabbit polyclonal anti-phospho-HACE1S385 (pS385)	N/A	This study*
Rabbit polyclonal anti-phospho-HACE1S337(pS337)	N/A	This study*
Polyclonal swine anti-mouse immunoglobulin HRP	N/A	DAKO
Polyclonal rabbit anti-rat immunoglobulin HRP	N/A	DAKO
Polyclonal goat anti-rabbit immunoglobulin HRP	N/A	DAKO

*See: methods

Acosta, Supplemental Figure S1

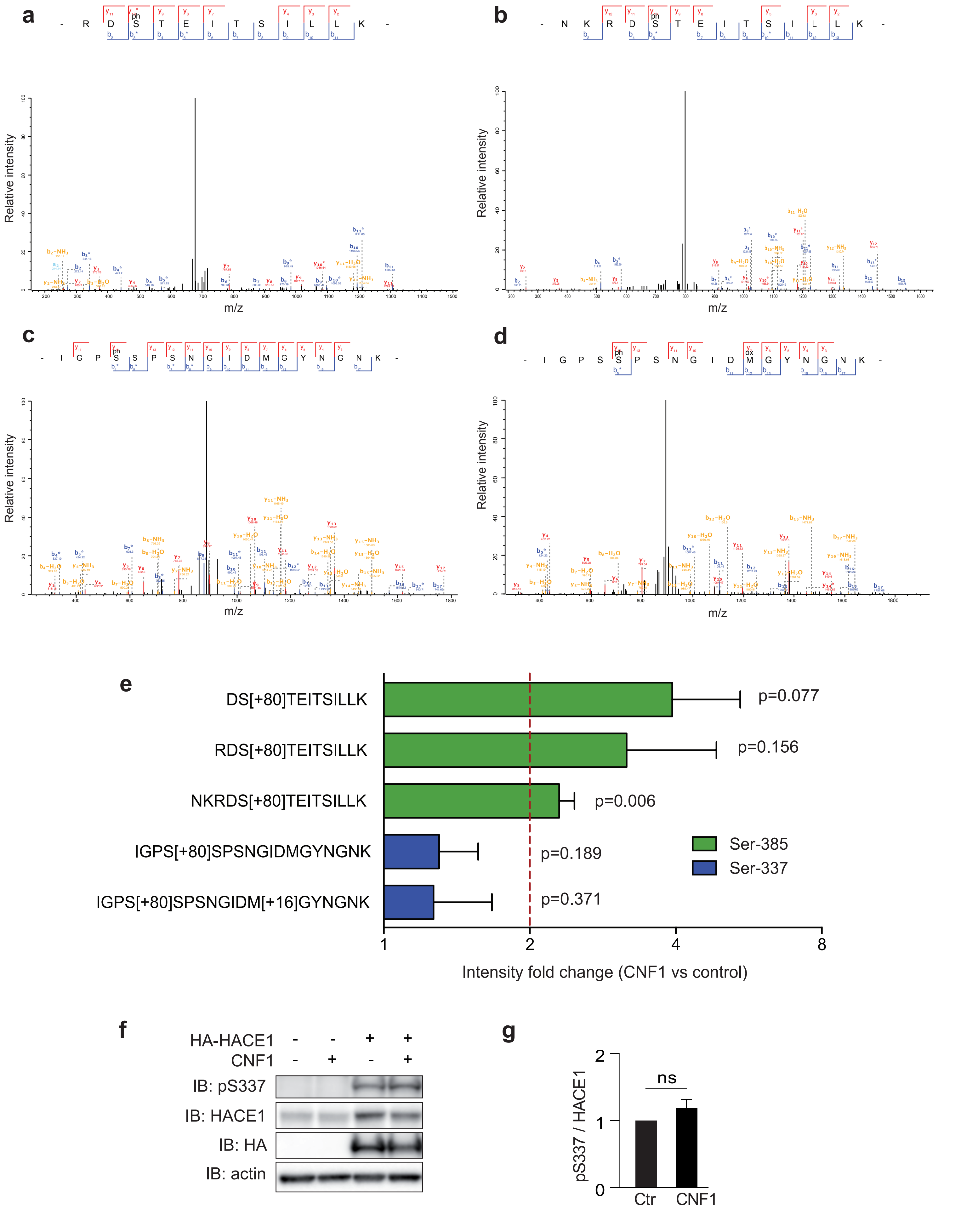

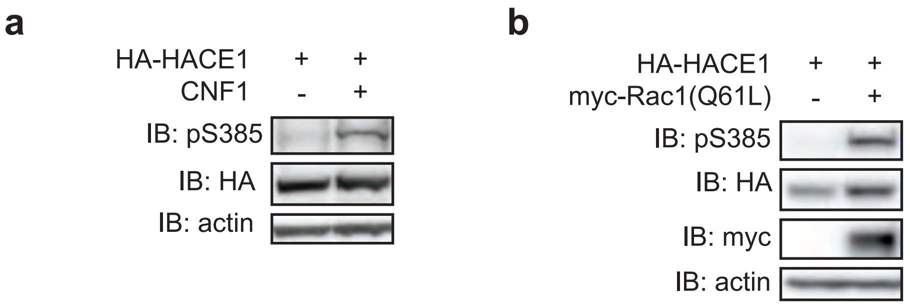
a
HA-HACE1 + +
CNF1 - +
IB: pS385
IB: HA
IB: actin
b
HA-HACE1 + +
myc-Rac1(Q61L) - +
IB: pS385
IB: HA
IB: myc
IB: actin

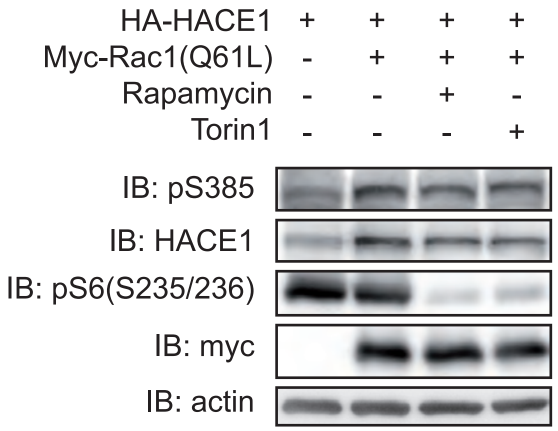
HA-HACE1 + + + +
Myc-Rac1(Q61L) - + + +
Rapamycin - - + -
Torin1 - - - +
IB: pS385
IB: HACE1
IB: pS6(S235/236)
IB: myc
IB: actin

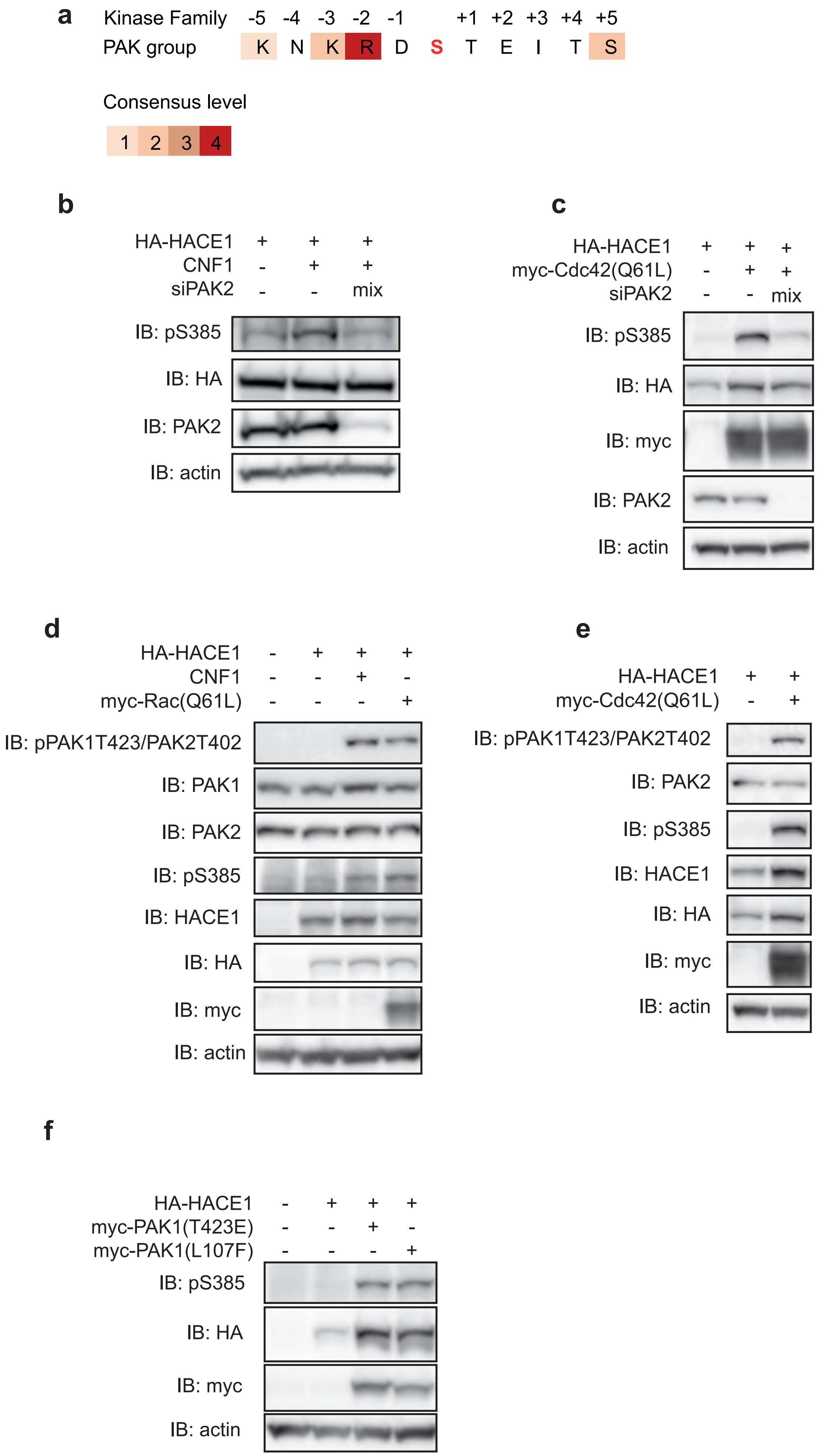
a
Kinase Family -5 -4 -3 -2 -1 +1 +2 +3 +4 +5
PAK group K N K R D S T E I T S
Consensus level
1 2 3 4
b
HA-HACE1 + + +
CNF1 - + +
siPAK2 - - mix
IB: pS385
IB: HA
IB: PAK2
IB: actin
c
HA-HACE1 + + +
myc-Cdc42(Q61L) - + +
siPAK2 - - mix
IB: pS385
IB: HA
IB: myc
IB: PAK2
IB: actin
d
HA-HACE1 - + + +
CNF1 - - + -
myc-Rac(Q61L) - - - +
IB: pPAK1T423/PAK2T402
IB: PAK1
IB: PAK2
IB: pS385
IB: HACE1
IB: HA
IB: myc
IB: actin
e
HA-HACE1 + +
myc-Cdc42(Q61L) - +
IB: pPAK1T423/PAK2T402
IB: PAK2
IB: pS385
IB: HACE1
IB: HA
IB: myc
IB: actin
f
HA-HACE1 - + + +
myc-PAK1(T423E) - - + -
myc-PAK1(L107F) - - - +
IB: pS385
IB: HA
IB: myc
IB: actin

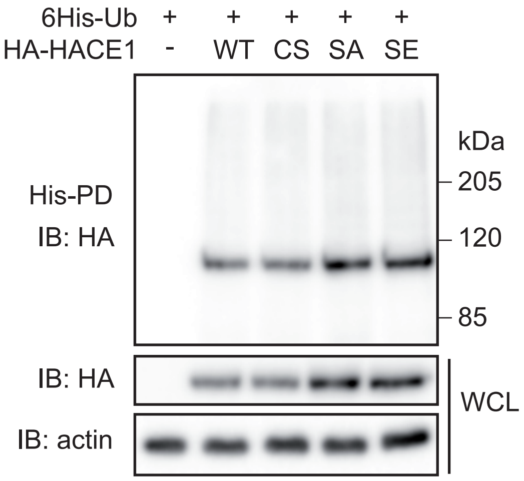

6His-Ub + + + + +
HA-HACE1 - WT CS SA SE
kDa
205
120
85
His-PD
IB: HA
IB: HA
IB: actin
WCL

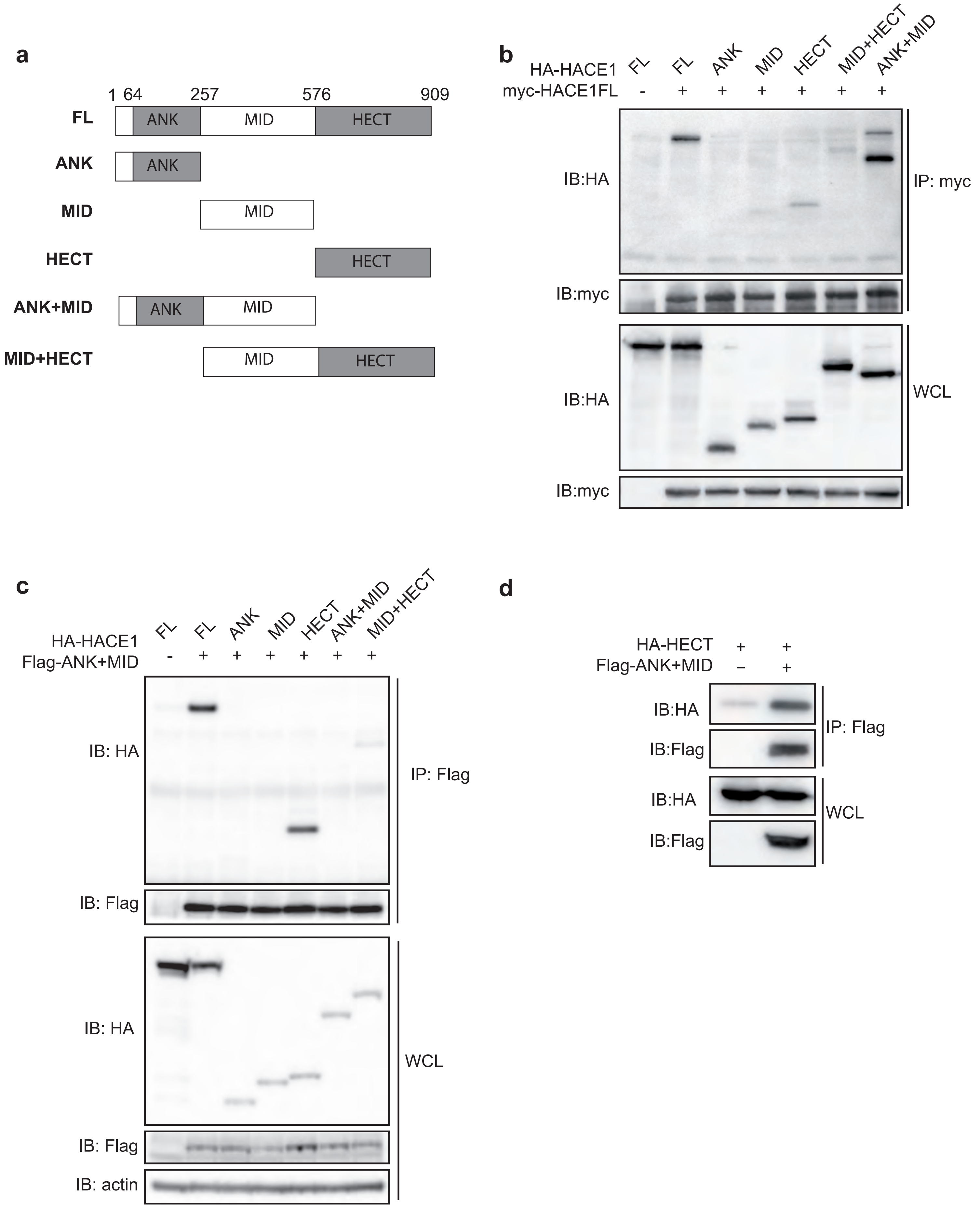
a
1 64
257
576
909
FL
ANK
MID
HECT
ANK+MID
MID+HECT
b
HA-HACE1
myc-HACE1FL
FL
ANK
MID
HECT
MID+HECT
ANK+MID
IB:HA
IB:myc
IP: myc
WCL
c
HA-HACE1
Flag-ANK+MID
IB: HA
IB: Flag
IB: actin
IP: Flag
WCL
d
HA-HECT
Flag-ANK+MID
IB:HA
IB:Flag
IP: Flag
WCL

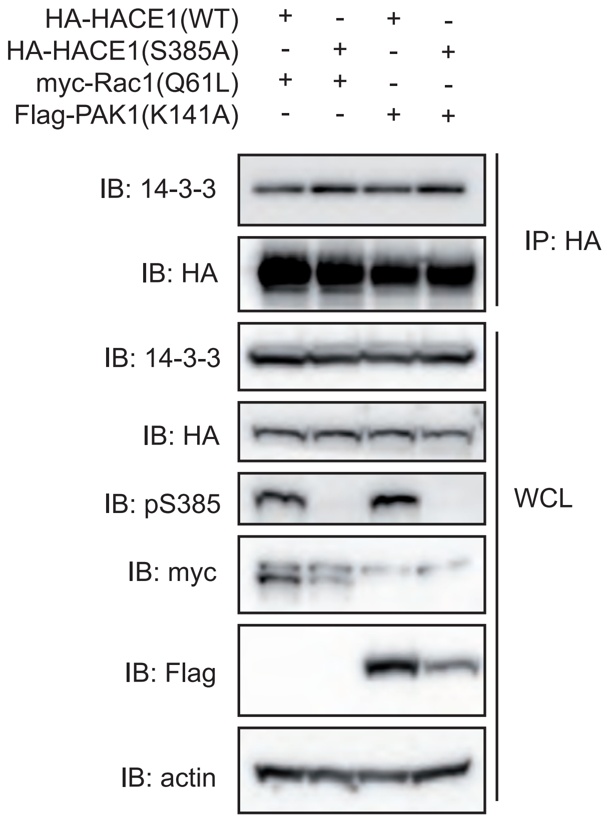
HA-HACE1(WT) + - + -
HA-HACE1(S385A) - + - +
myc-Rac1(Q61L) + + - -
Flag-PAK1(K141A) - - + +
IB: 14-3-3
IB: HA
IP: HA
IB: 14-3-3
IB: HA
IB: pS385
IB: myc
IB: Flag
IB: actin
WCL

Acosta, Supplementary Figure S8

a

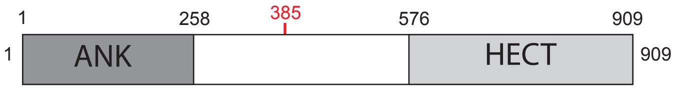

b

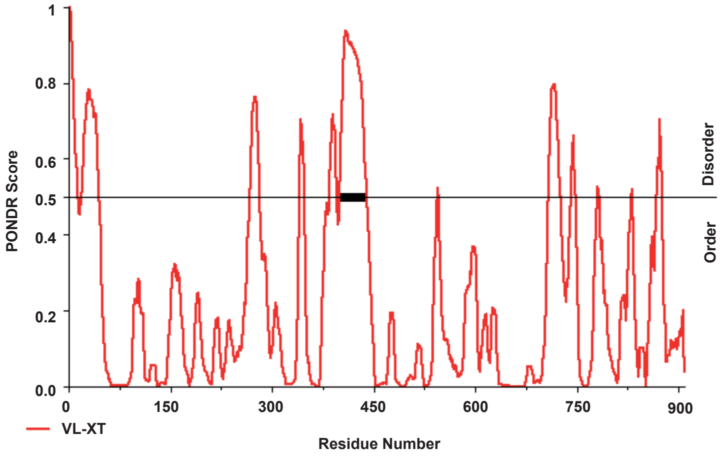

c

VL-XT predicted disorder segment				
IDR	starting aa	ending aa	size	average strength
1	1	13	13	0.7777
2	19	45	27	0.681
3	267	281	15	0.6685
4	341	347	7	0.6339
5	385	395	11	0.6343
6	400	439	40	0.8115
7	544	545	2	0.5146
8	708	725	18	0.6925
9	741	747	7	0.5837
10	781	782	2	0.5193
11	831	831	1	0.5208
12	868	875	8	0.5907

d

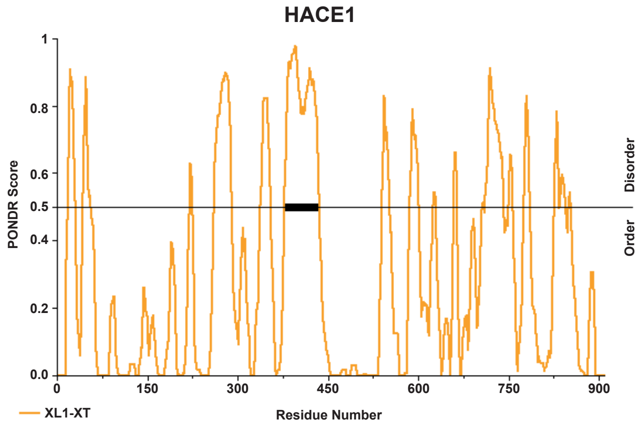

e

XL1-XT predicted disorder segment				
IDR	starting aa	ending aa	size	average strength
1	18	30	11	0.7369
2	44	56	11	0.6615
3	220	225	4	0.5783
4	261	289	27	0.7900
5	338	355	16	0.7059
6	378	436	57	0.8320
7	541	552	10	0.6923
8	587	600	12	0.6830
9	625	629	3	0.5317
10	659	664	4	0.6249
11	707	709	1	0.5061
12	711	741	29	0.7218
13	750	757	6	0.6007
14	776	785	8	0.6859
15	826	833	6	0.6519
16	835	844	8	0.5509
17	850	852	1	0.5222

f

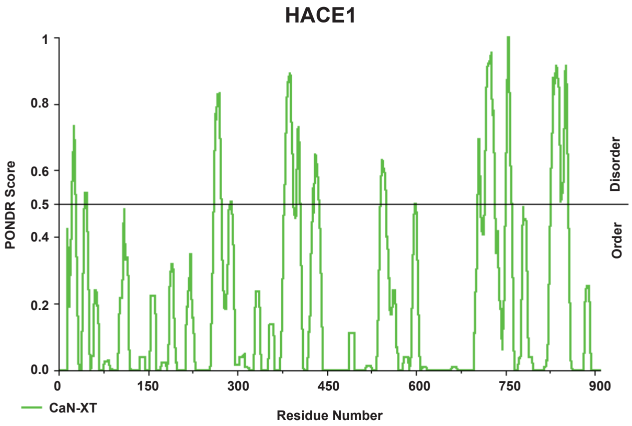

g

CaN-XT predicted disorder segment				
IDR	starting aa	ending aa	size	average strength
1	23	28	6	0.6312
2	44	47	4	0.5311
3	261	273	13	0.7262
4	288	291	4	0.5065
5	379	393	15	0.7682
6	400	405	6	0.6402
7	427	427	1	0.5442
8	429	437	9	0.5954
9	541	549	9	0.6014
10	598	600	3	0.5001
11	704	708	5	0.6257
12	715	733	19	0.7890
13	751	760	10	0.7930
14	827	855	29	0.7409

Acosta, Supplementary Figure S9

Figure 1b

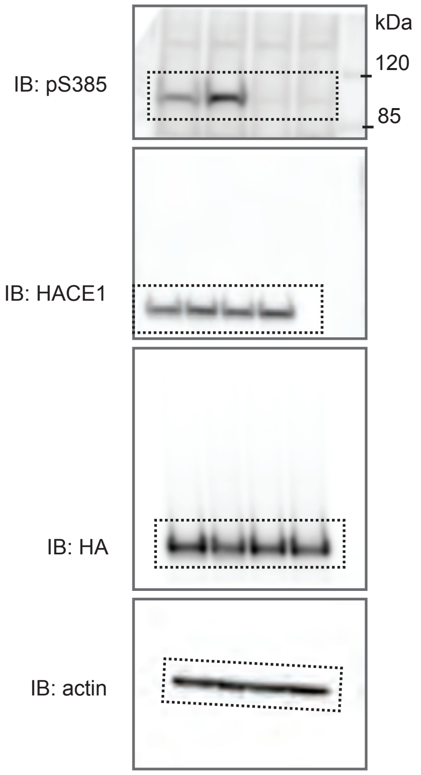

Figure 1d

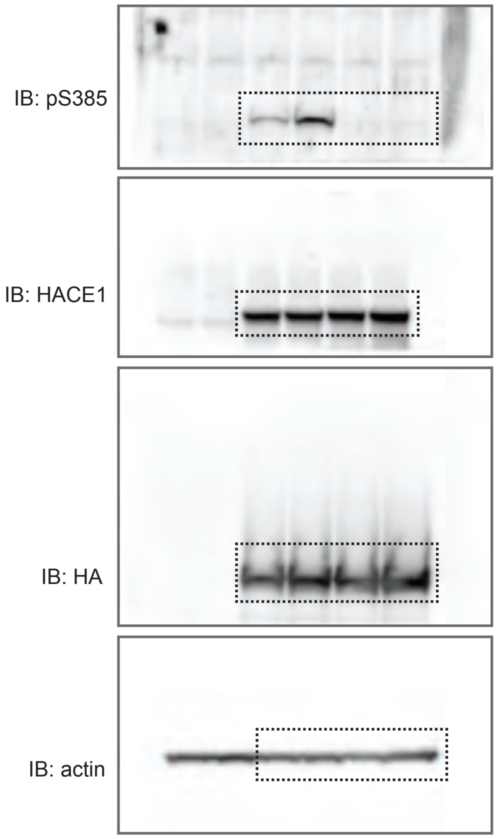

Figure 1e

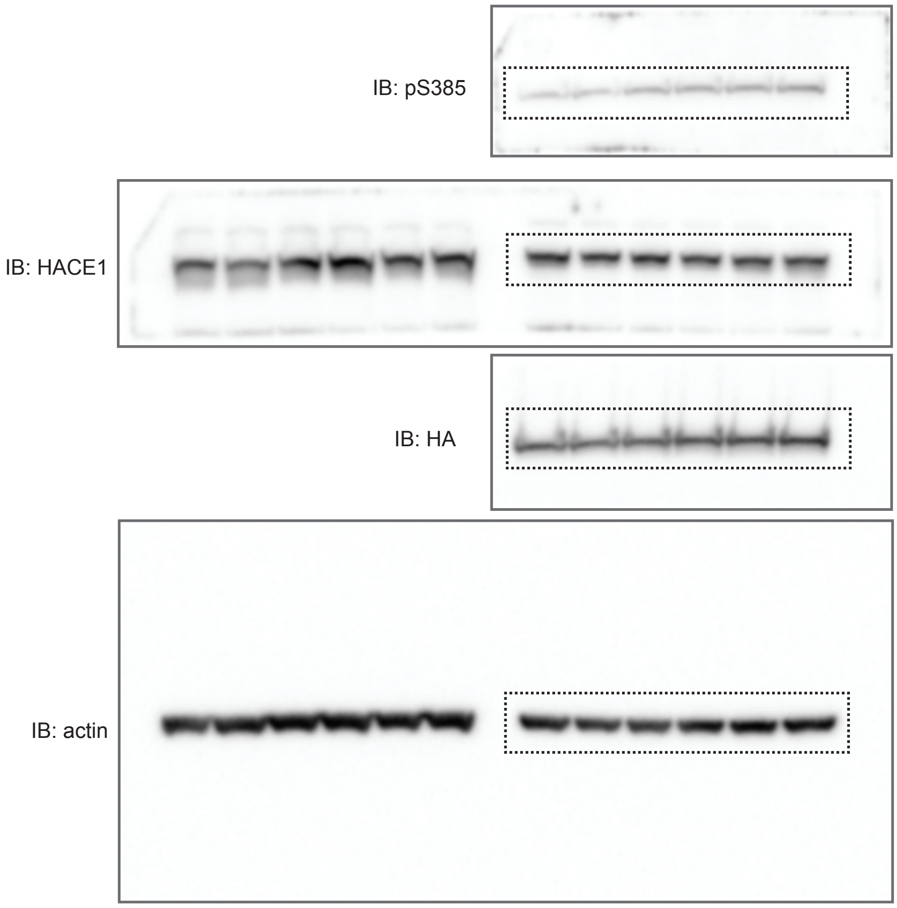

Figure 1f

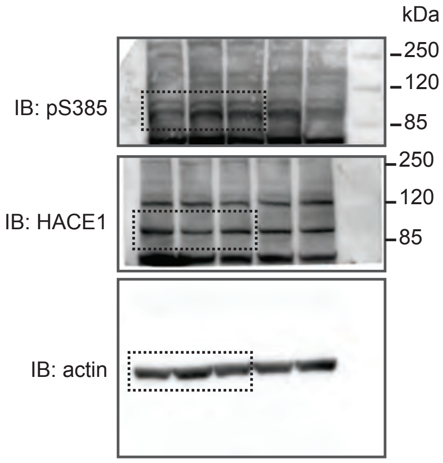

Figure 2a

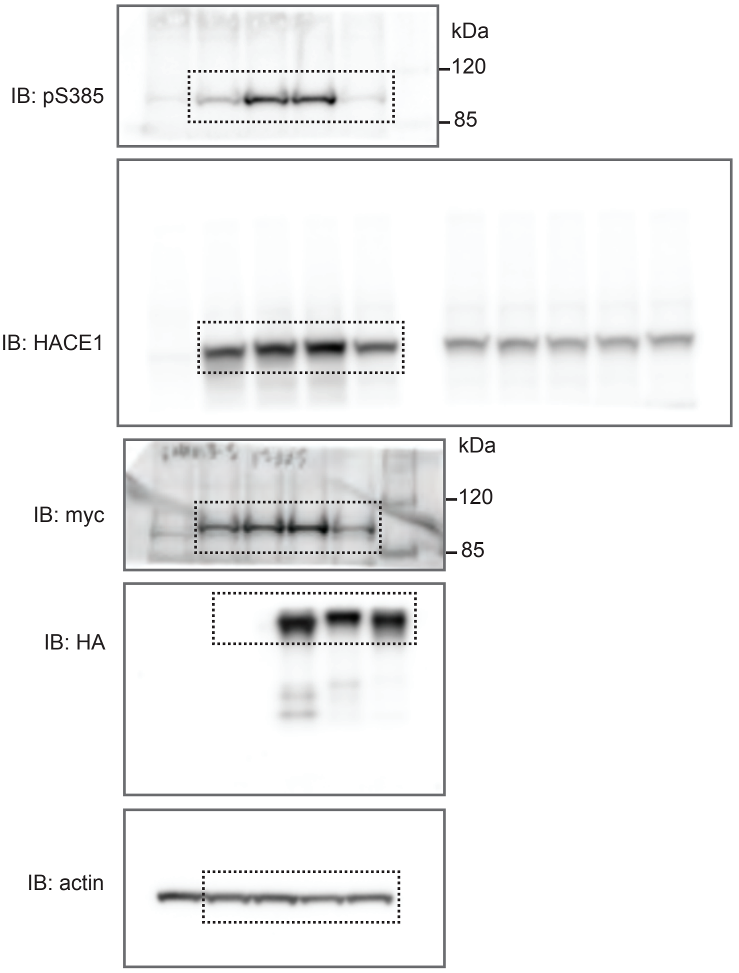

Figure 2c

Figure 2f

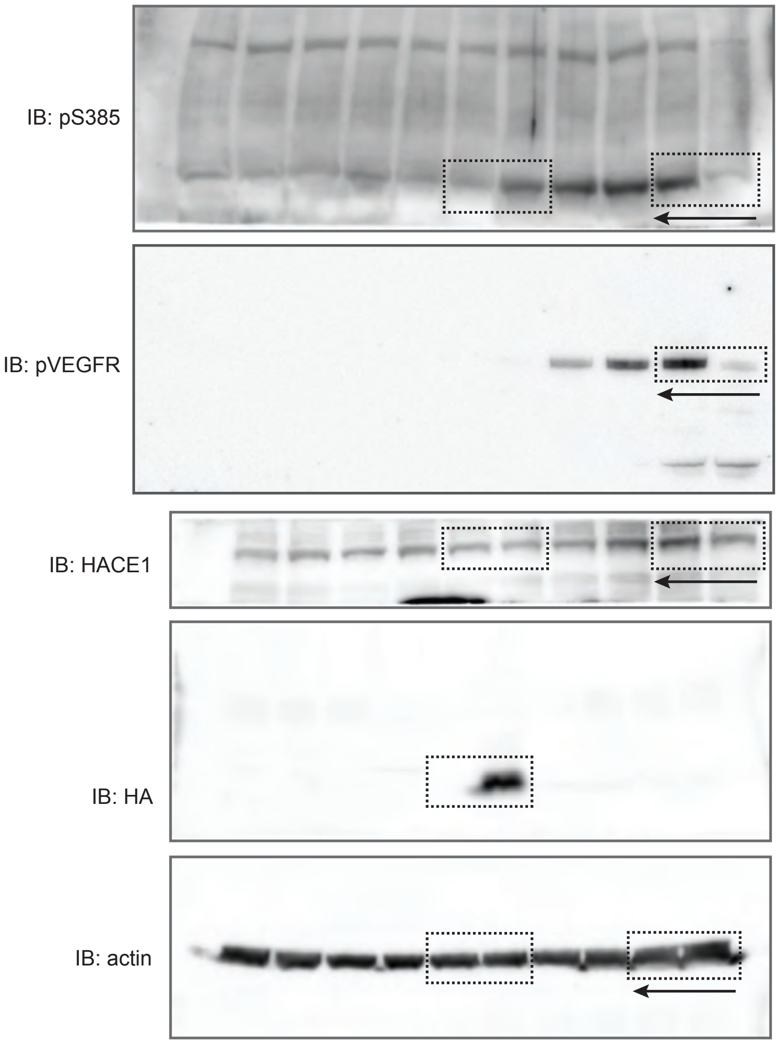

Figure 2d

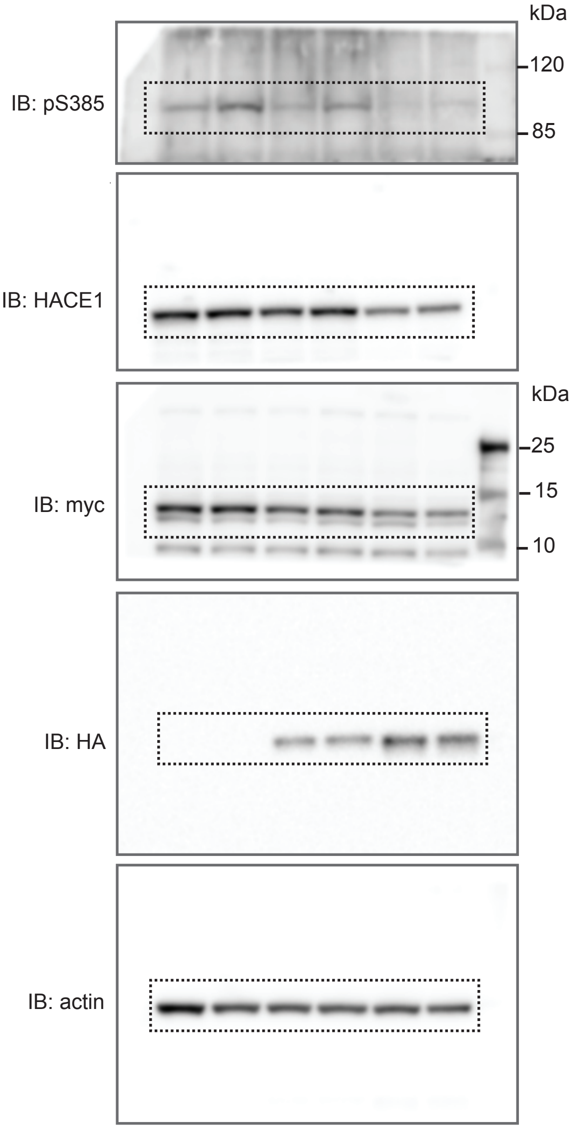

Acosta, Supplementary Figure S11

Figure 3a

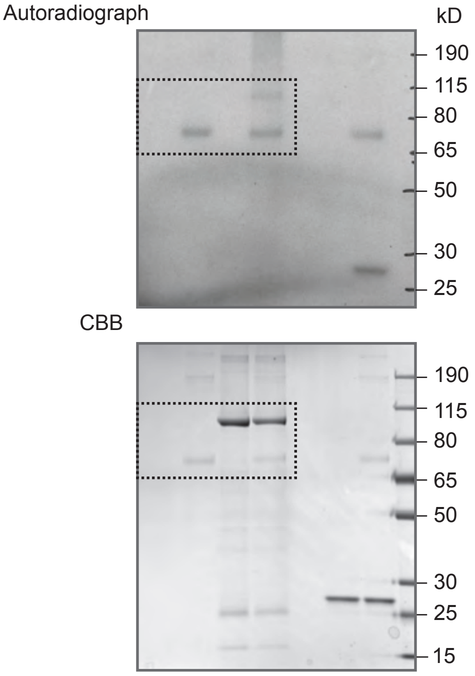

Figure 3b

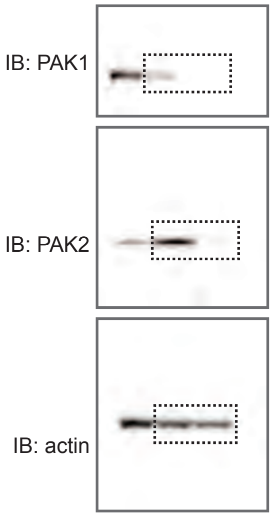

Figure 3c

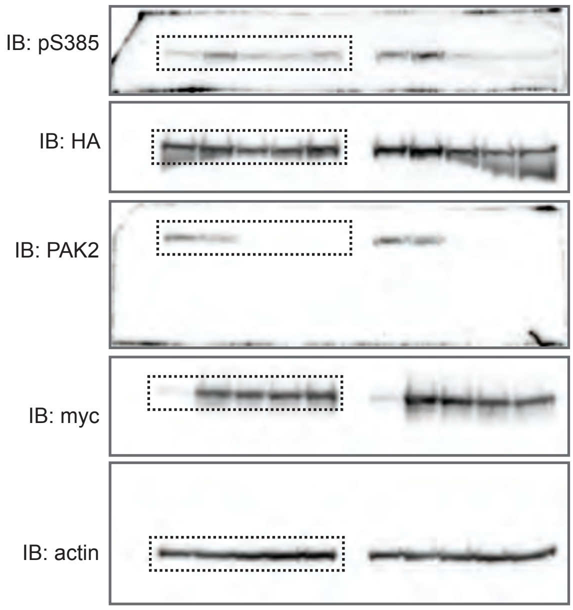

Figure 3d

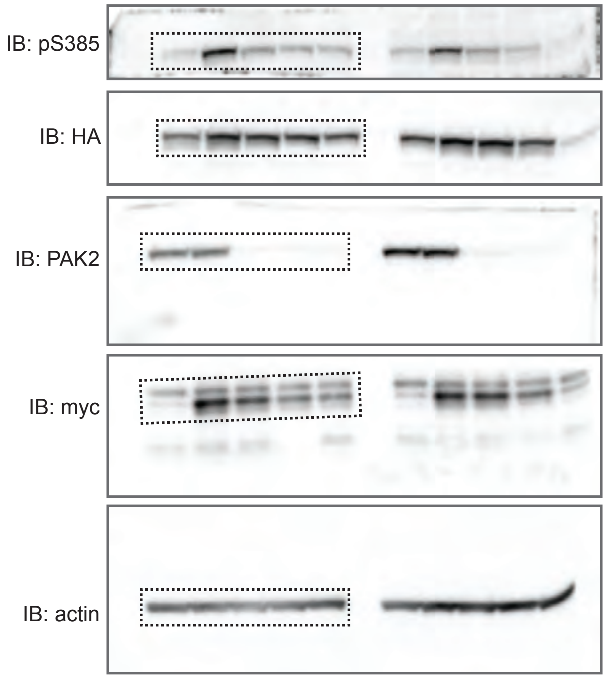

Figure 3e

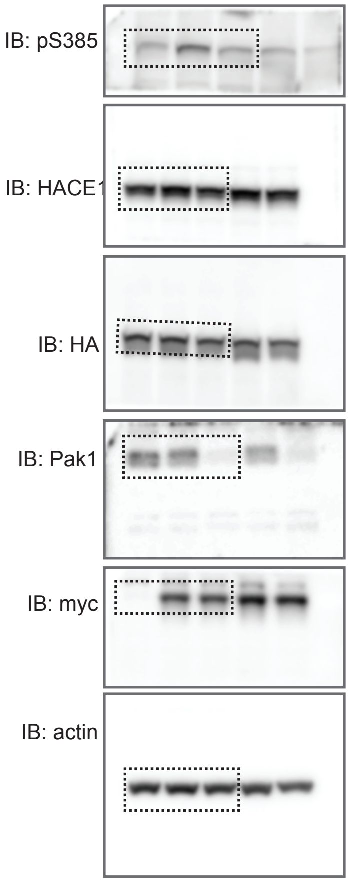

Figure 3f

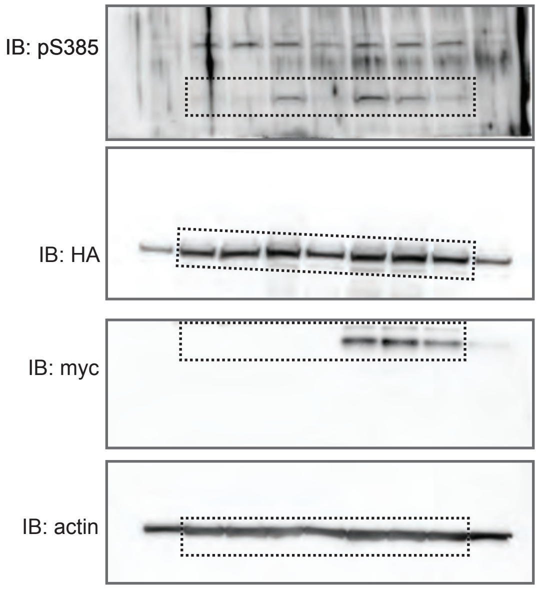

Figure 3g

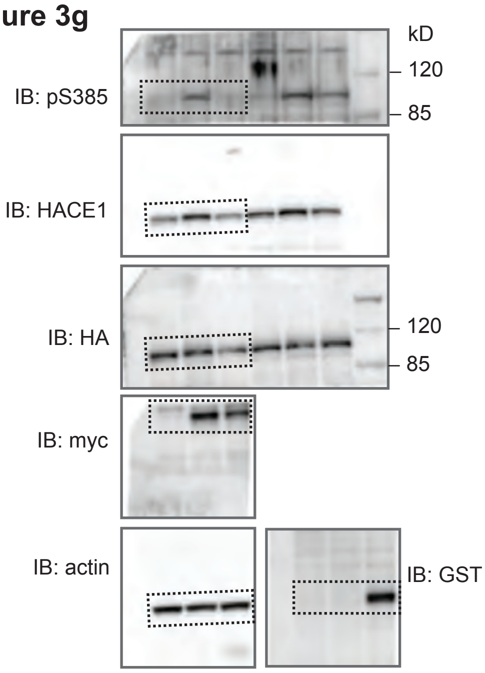

Figure 3h

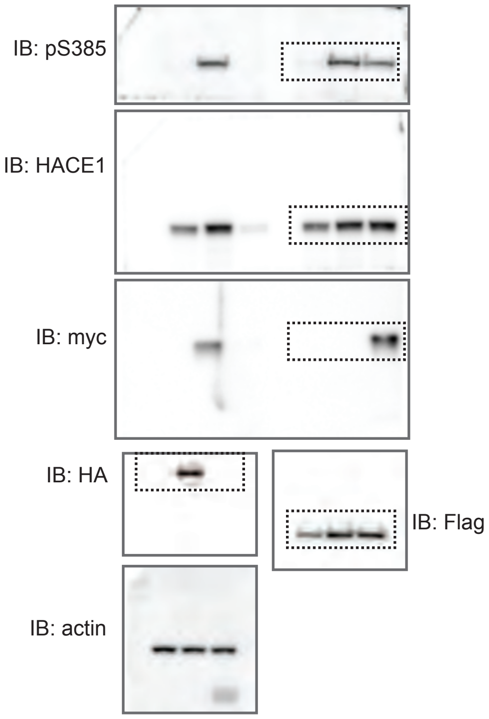

Figure 4a

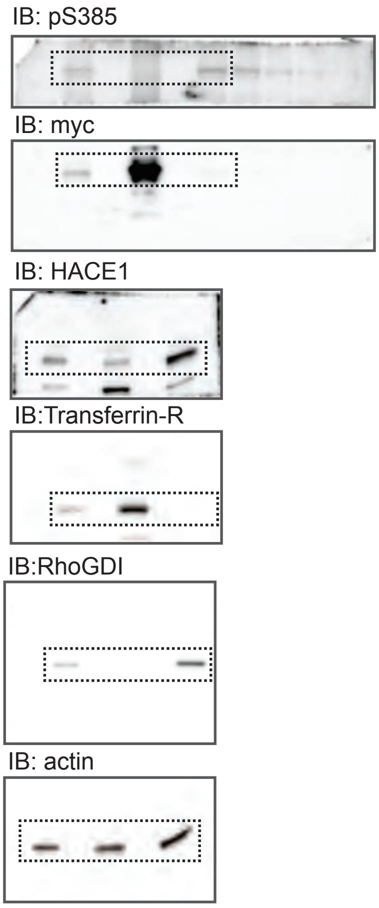

Figure 4b

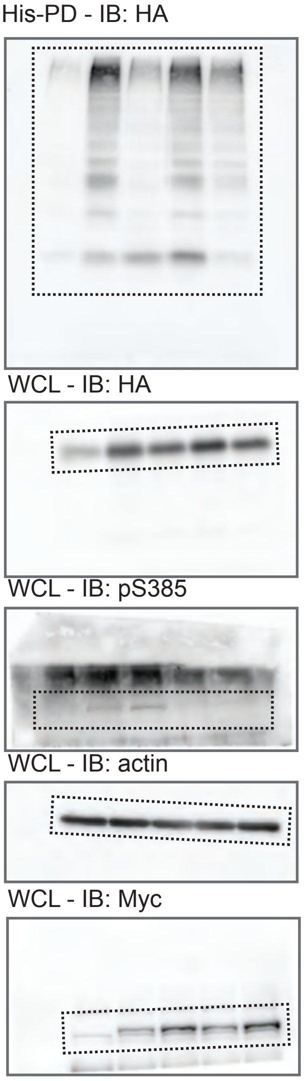

Figure 4c

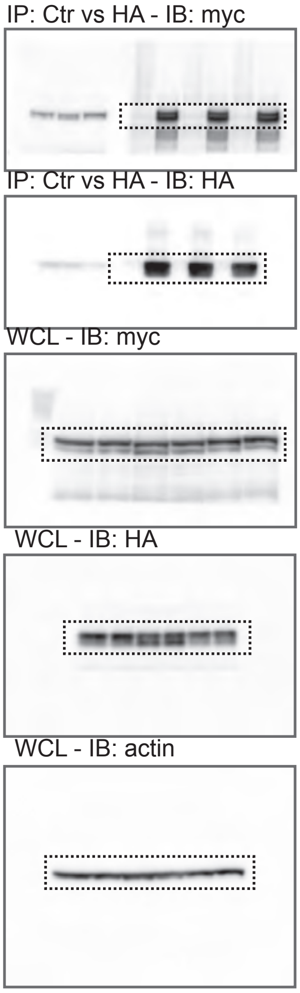

Figure 4d

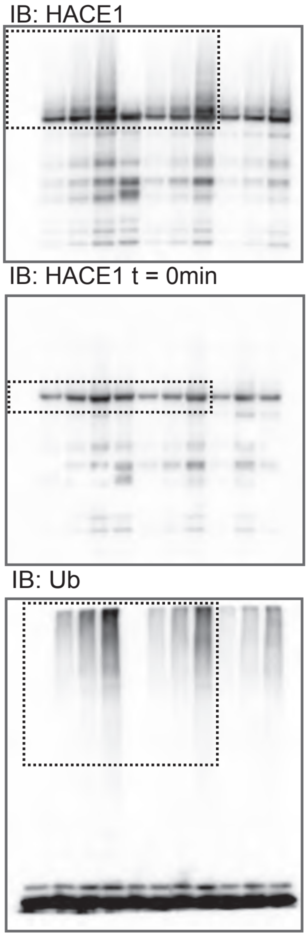

Figure 4e

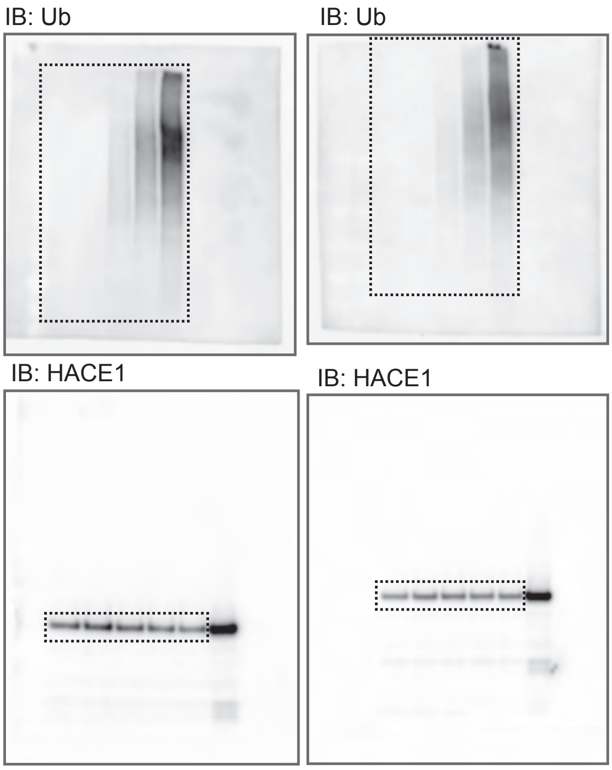

Figure 4f

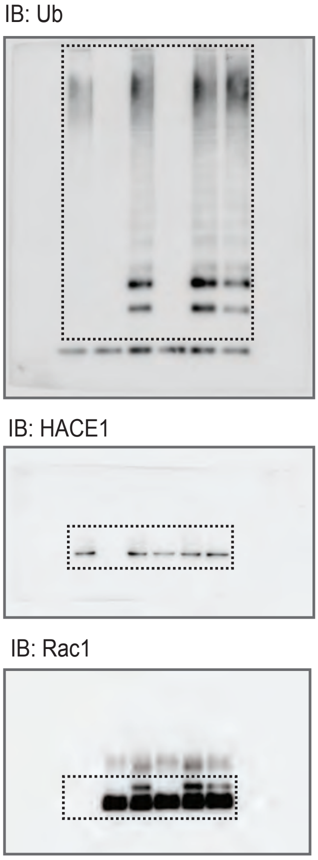

Figure 5a

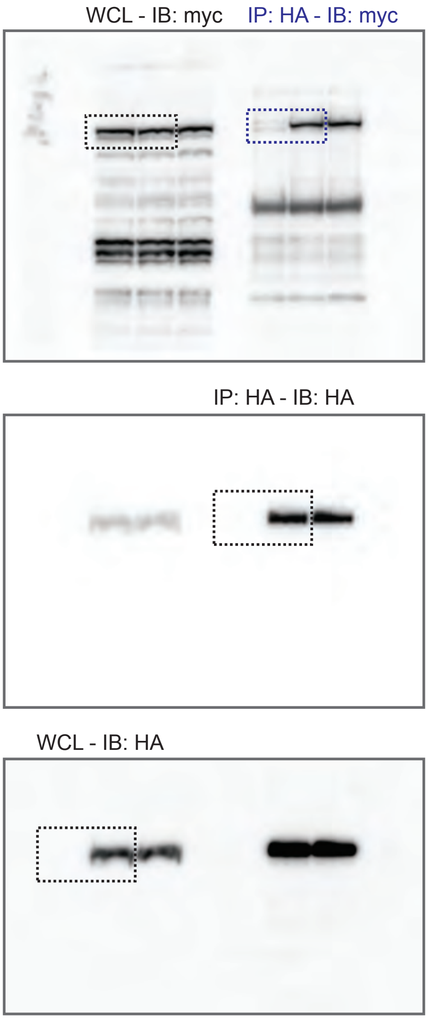

Figure 5d

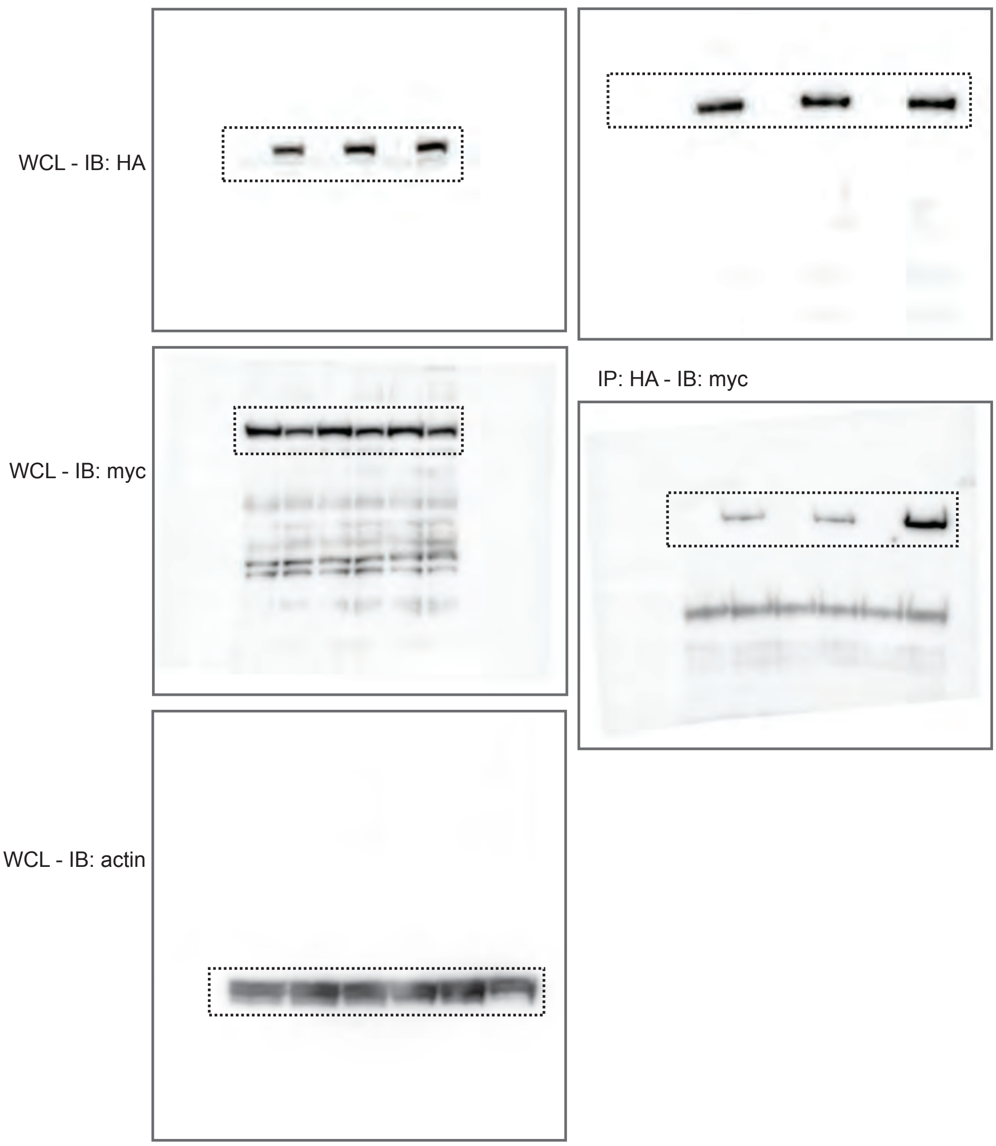

Figure 5e

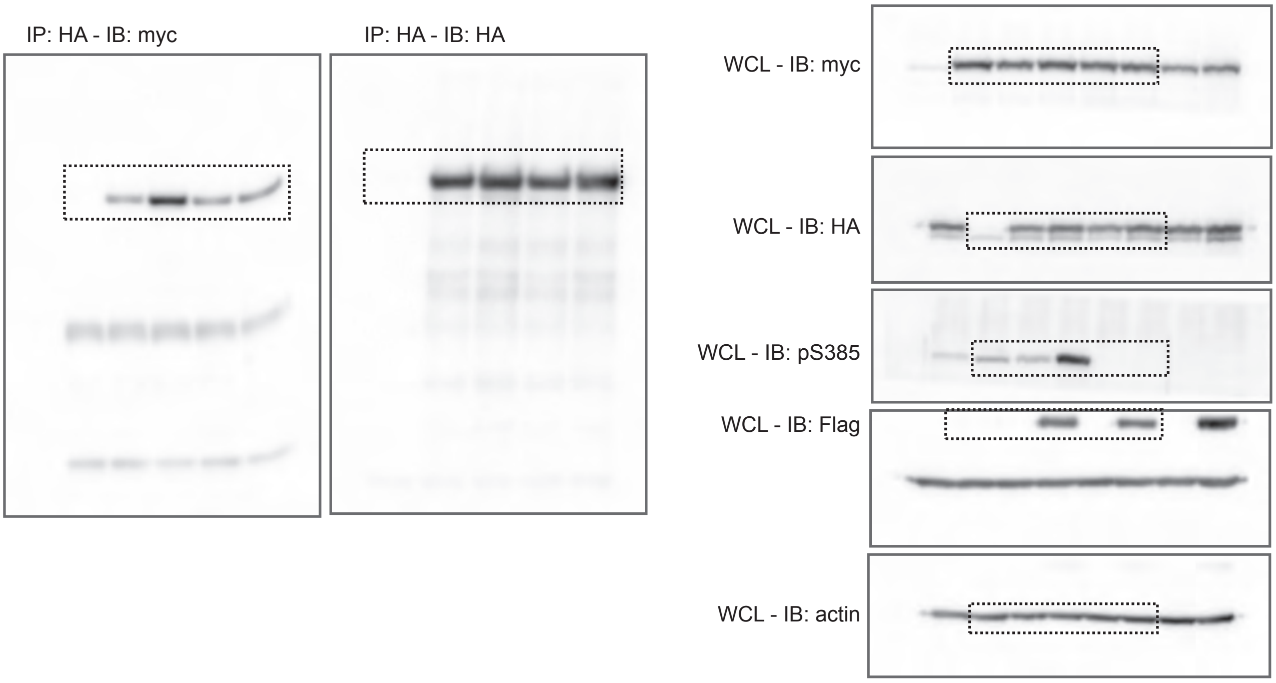

Supplement: Supplementary file 1 — Supplementary Information [file 41598_2018_19471_MOESM1_ESM.pdf]
